# Monitoring Central Venous Catheter Resistance to Predict Imminent Occlusion: A Prospective Pilot Study

**DOI:** 10.1371/journal.pone.0135904

**Published:** 2015-08-31

**Authors:** Joshua Wolf, Li Tang, Jeffrey E. Rubnitz, Rachel C. Brennan, David R. Shook, Dennis C. Stokes, Paul Monagle, Nigel Curtis, Leon J. Worth, Kim Allison, Yilun Sun, Patricia M. Flynn

**Affiliations:** 1 Department of Infectious Diseases, St. Jude Children’s Research Hospital, Memphis, Tennessee, United States of America; 2 Department of Biostatistics, St. Jude Children’s Research Hospital, Memphis, Tennessee, United States of America; 3 Department of Oncology, St. Jude Children’s Research Hospital, Memphis, Tennessee, United States of America; 4 Department of Bone Marrow Transplantation and Cellular Therapy, St. Jude Children’s Research Hospital, Memphis, Tennessee, United States of America; 5 Department of Pediatrics, University of Tennessee Health Science Center, Memphis, Tennessee, United States of America; 6 Department of Haematology, Royal Children’s Hospital, Parkville, Victoria, Australia; 7 Murdoch Children’s Research Institute, Parkville, Victoria, Australia; 8 Infectious Diseases Unit, The Royal Children’s Hospital Melbourne, Parkville, Victoria, Australia; 9 Peter MacCallum Cancer Centre, East Melbourne, Victoria, Australia; 10 Department of Paediatrics, University of Melbourne, Parkville, Victoria, Australia; 11 Department of Medicine, University of Melbourne, Parkville, Victoria, Australia; Ohio State University, UNITED STATES

## Abstract

**Background:**

Long-term central venous catheters are essential for the management of chronic medical conditions, including childhood cancer. Catheter occlusion is associated with an increased risk of subsequent complications, including bloodstream infection, venous thrombosis, and catheter fracture. Therefore, predicting and pre-emptively treating occlusions should prevent complications, but no method for predicting such occlusions has been developed.

**Methods:**

We conducted a prospective trial to determine the feasibility, acceptability, and efficacy of catheter-resistance monitoring, a novel approach to predicting central venous catheter occlusion in pediatric patients. Participants who had tunneled catheters and were receiving treatment for cancer or undergoing hematopoietic stem cell transplantation underwent weekly catheter-resistance monitoring for up to 12 weeks. Resistance was assessed by measuring the inline pressure at multiple flow-rates via a syringe pump system fitted with a pressure-sensing transducer. When turbulent flow through the device was evident, resistance was not estimated, and the result was noted as “non-laminar.”

**Results:**

Ten patients attended 113 catheter-resistance monitoring visits. Elevated catheter resistance (>8.8% increase) was strongly associated with the subsequent development of acute catheter occlusion within 10 days (odds ratio = 6.2; 95% confidence interval, 1.8–21.5; p <0.01; sensitivity, 75%; specificity, 67%). A combined prediction model comprising either change in resistance greater than 8.8% or a non-laminar result predicted subsequent occlusion (odds ratio = 6.8; 95% confidence interval, 2.0–22.8; p = 0.002; sensitivity, 80%; specificity, 63%). Participants rated catheter-resistance monitoring as highly acceptable.

**Conclusions:**

In this pediatric hematology and oncology population, catheter-resistance monitoring is feasible, acceptable, and predicts imminent catheter occlusion. Larger studies are required to validate these findings, assess the predictive value for other clinical outcomes, and determine the impact of pre-emptive therapy.

**Trial Registration:**

Clinicaltrials.gov NCT01737554

## Introduction

Medium- and long-term central venous catheters (CVCs) are essential to modern medical practice. These devices may remain in situ for months or years during therapy, and serious complications, including bloodstream infection, venous thrombosis, and occlusion are frequent [1,2]. CVC occlusion occurs at a rate of at least 2.0 to 2.8 events/1000 CVC days and affects as many as 36% of patients [1,3,4]. The mechanisms underlying occlusion are not well understood, but malposition, fibrin sheaths, intraluminal deposition of drugs, parenteral nutrition, thrombus, and bacterial biofilm are all thought to play a role [3,5]. These events may occur rapidly or gradually over time.

Occlusion is a substantial impediment to delivering care, because it interferes with the normal use of the CVC and can lead to drug extravasation, device removal, or other serious complications [1,3,5,6]. Additionally, occlusion is strongly associated with subsequent bloodstream infection, venous thrombosis, catheter fracture, and even death [1,7–10]. Therefore, the successful prediction and pre-emptive treatment of occlusions should help prevent these life-threatening complications.

Resistance to laminar flow through a tube is inversely related to the diameter of the lumen, and previous data suggest that CVC flow can be laminar [11,12]. Because narrowing of a CVC lumen can occur before complete occlusion, catheter resistance may be measurably elevated when occlusion is imminent but not yet clinically apparent [13]. To further explore this possibility, we undertook a prospective study of serial catheter-resistance monitoring (CRM) that involved weekly estimations of resistance to flow through the device to determine the feasibility, acceptability, and efficacy of this novel approach.

## Patients and Methods

### Study population

This pilot study was performed between January and December 2013, at St. Jude Children’s Research Hospital. Eligible participants were patients 5 to 25 years of age who were receiving treatment that necessitated weekly hospital visits and had a tunneled CVC (Hickman Catheter, Bard Access Systems, Salt Lake City, UT, USA), which was expected to remain in situ for at least 12 weeks. Potential participants were approached during regular hospital visits or inpatient stays based on notifications from treating clinicians.

**Fig 1:**
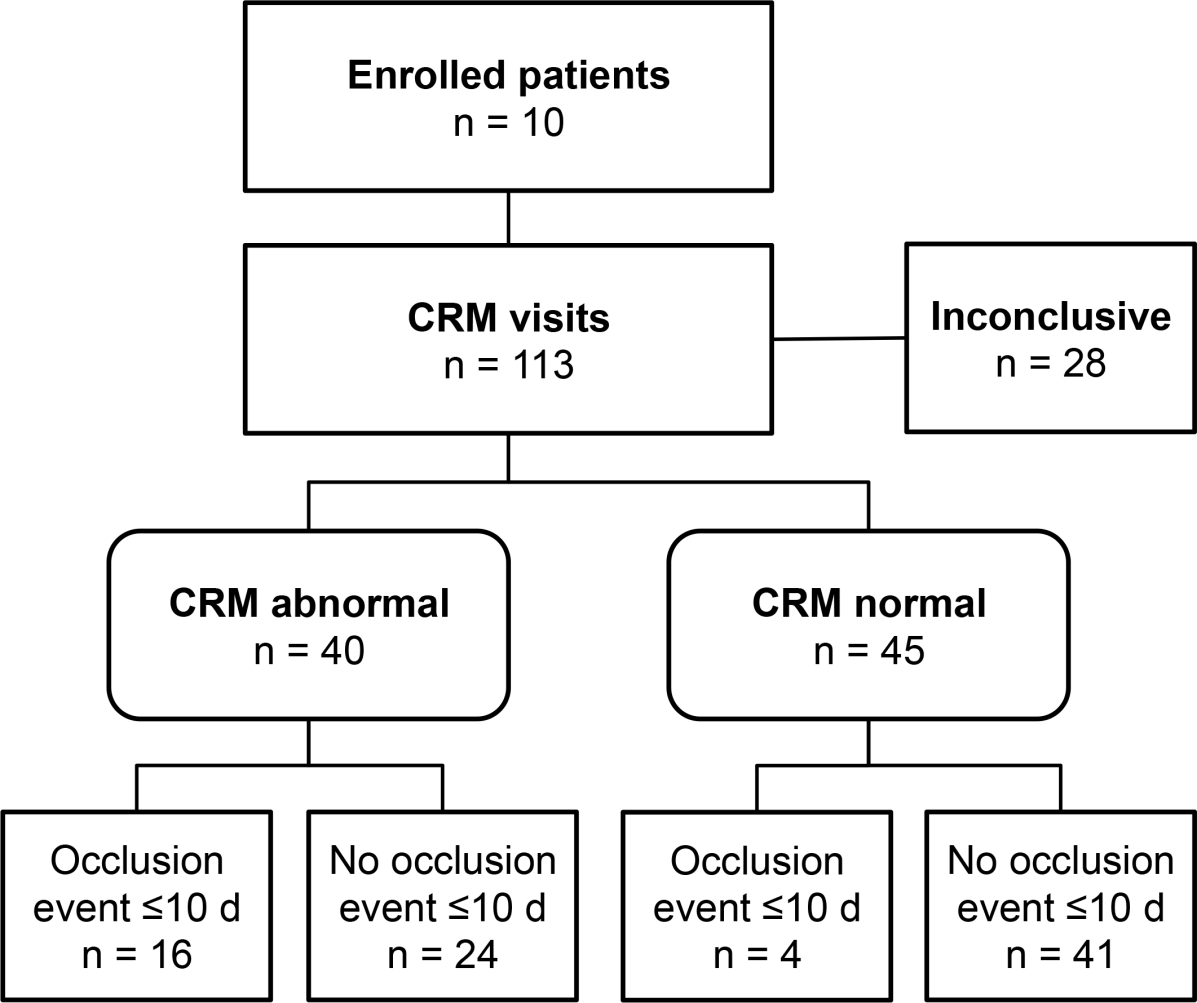
Flow diagram of CRM results and clinical outcomes. Per the STARD (STAndards for Reporting Diagnostic accuracy studies) initiative, the design of the study and flow of the patients are diagrammed [14]. The reasons for inconclusive CRM included first visit (n = 10), previous visit *R* ^*2*^ was less than 85% (n = 7), TPA since previous visit (n = 10), and abnormal CVC function (n = 1). “CRM normal” indicates that the change in resistance was less than 8.8%. “CRM abnormal” indicates that either the change in resistance was at least 8.8% or the *R* ^*2*^ was less than 85%. Clinical outcomes are also noted.

The following baseline data were obtained for each participant: demographic details, characteristics of primary disease, and CVC specifics (i.e., type, size, anatomic location, insertion date, and previous complications). Additional data were prospectively collected weekly for as long as 15 weeks: attendance at study visits, reasons for missed visits, clinically apparent occlusion or administration of tissue plasminogen activator (TPA) for CVC occlusion, and occurrence of all other clinically significant CVC-related complications [i.e., catheter fracture, central line–associated bloodstream infection (CLABSI), or venous thrombosis]. Investigators collecting data about these outcomes were not blind to CRM results, but clinicians who diagnosed clinical events and prescribed therapy were blind to CRM results. This report uses the format of the Standards for Reporting Diagnostic Accuracy Initiative (Fig 1).

There were a total of 113 CRM visits for the 10 patients studied. Of these, 28 visits were excluded for the following reasons: first visit (n = 10), previous visit *R* ^*2*^ less than 85% (n = 7), TPA since previous visit (n = 10), and clinically abnormal CVC function (n = 1). Excluded CRM visits were termed “inconclusive”. In Fig 1, “CRM normal” (n = 45) indicates that the change in resistance was less than 8.8% and “CRM abnormal” (n = 40) indicates that either the change in resistance was at least 8.8% or the *R* ^*2*^ was less than 85%. Clinical outcomes are detailed in Fig 1.

### Definitions

*Occlusions* were classified by the lumen involved [red (larger lumen), white (smaller lumen), or both], event type (total occlusion, occlusion to flush only, occlusion to aspiration only, or subjective difficulty with flushing or aspiration only), and treatment required (spontaneous resolution, TPA, or CVC removal). Treatment of CVC occlusion was at the discretion of the treating clinician. Occlusion was not deemed present if external mechanical obstruction (e.g., kinking of the external CVC components) was evident. *Venous thrombosis* was defined by radiologic confirmation of partial or complete venous obstruction. *CLABSI* was defined according to the National Healthcare Safety Network surveillance definition [15]. *Adherence* was defined as the proportion of planned CRM visits that were attended. *Feasibility* was defined as the proportion of attended visits that provided usable CRM data (i.e., pressure measurements obtained for at least 3 flow rates for each lumen of the CVC).

### Catheter-resistance monitoring

**Fig 2:**
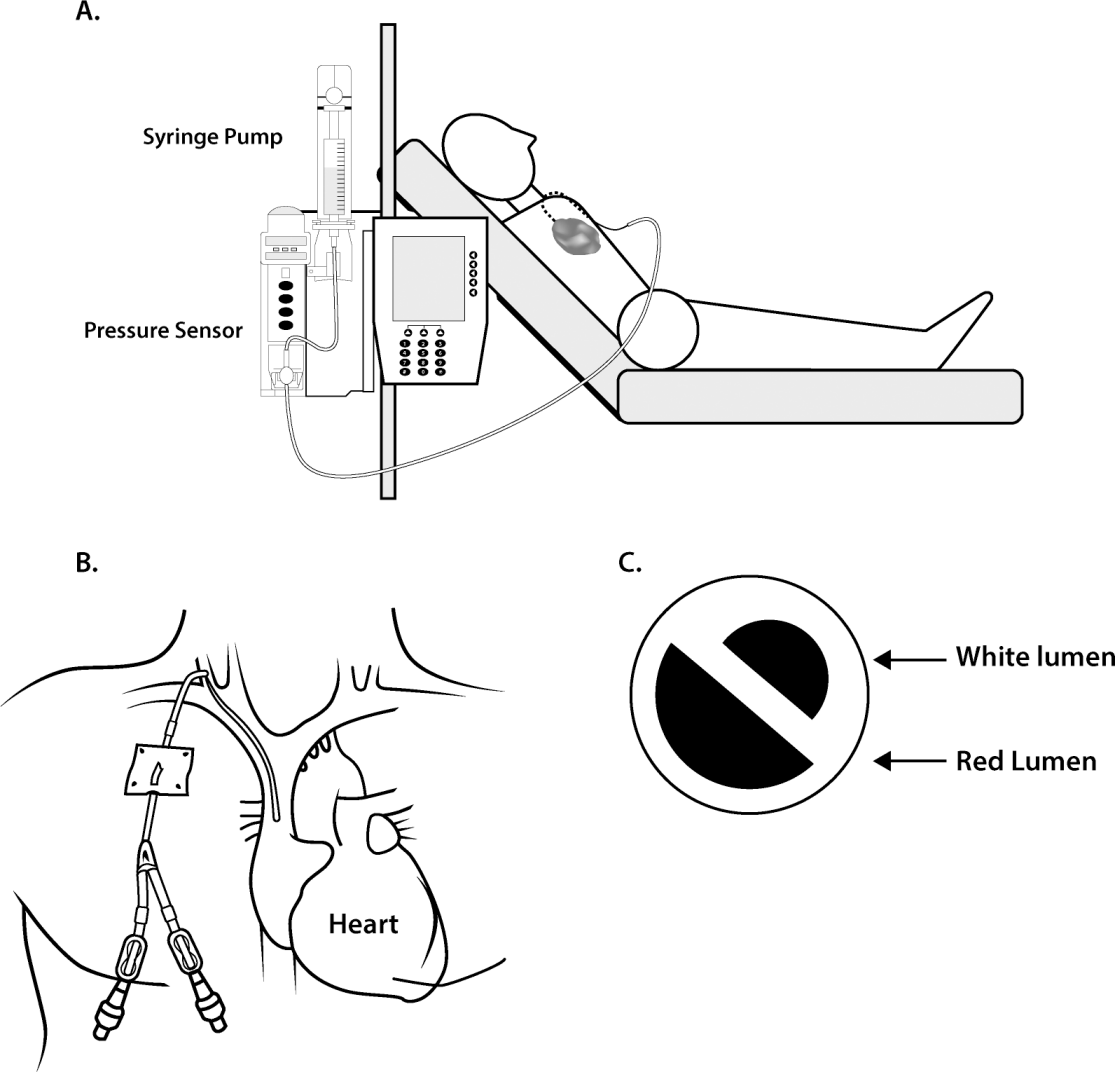
Patient positioning during CRM. (A) Resistance measurements were performed with the participant lying at an angle of approximately 45°. The pressure sensor was placed below the estimated height of the participant’s right atrium. The relative heights and positions of the patient and pump were not altered between or during measurements. (B) Illustration of a tunneled external CVC. (C) Diagram of the catheter cross-section showing the different luminal diameters.

**Fig 3:**
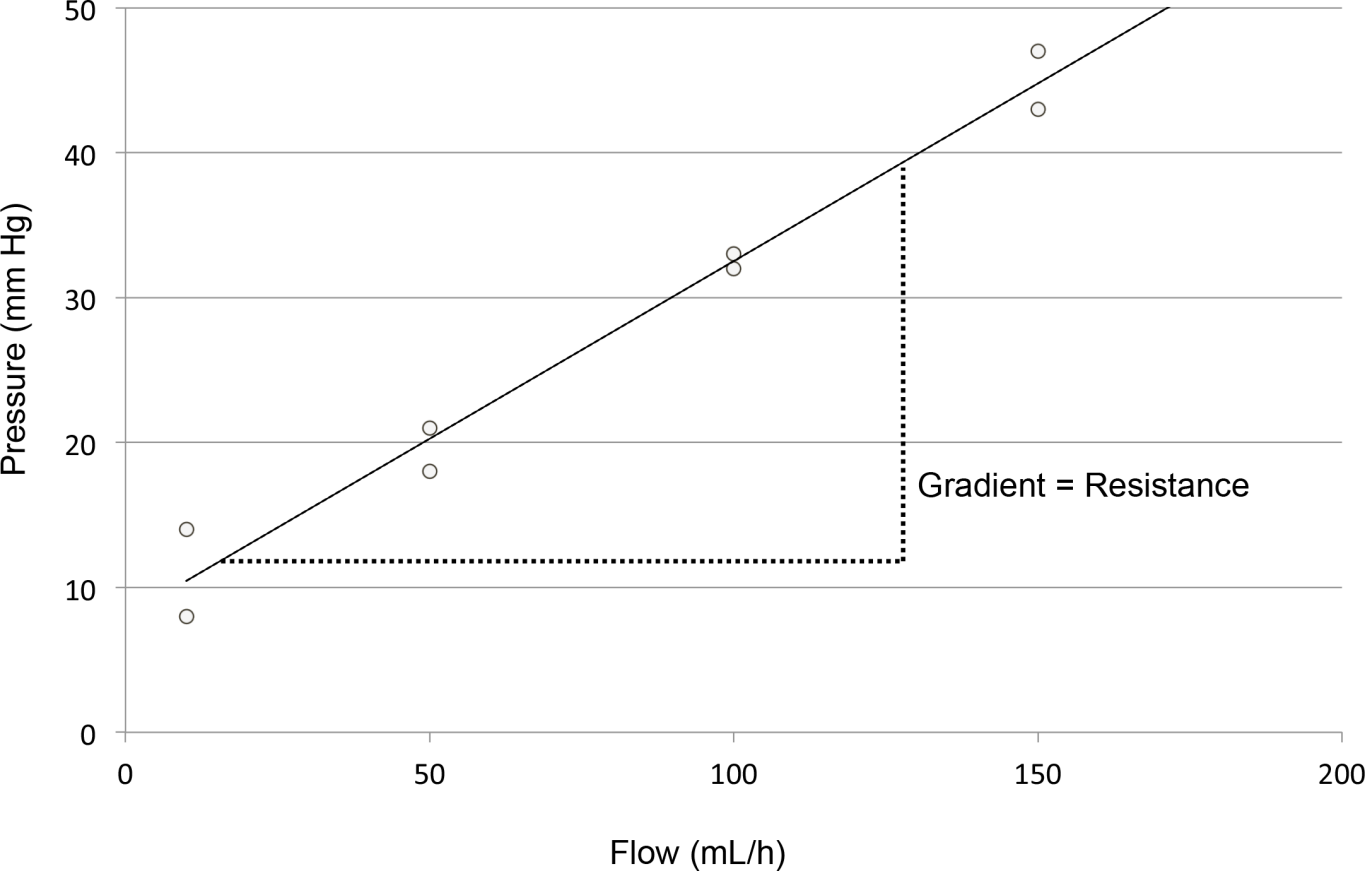
Estimation of catheter resistance. Resistance was calculated as the gradient of a pressure-flow plot. Results that were a poor fit to a least squares regression line (*R* ^*2*^ <85%) were termed non-laminar, and resistance was not estimated.

CRM was performed weekly by trained study staff (medical doctor or registered nurse) for up to 12 weeks, and participants were followed for clinical endpoints for 3 weeks after completion of the final CRM visit. During CRM visits, normal saline was infused through each lumen of the CVC at various predetermined flow rates while inline pressure was measured. Specifically, staff measured the inline pressure in the CVC twice at each of 4 flow rates (typically 10, 50, 100, and 150 mL/h). Pressure was measured using a syringe pump with an incorporated pressure-sensing transducer (Alaris 8110 Syringe Pump, CareFusion Inc., San Diego, CA). The pressure sensor was placed below the estimated height of the participant’s right atrium, with the participant lying at an angle of approximately 45°**.** The relative heights and positions of the patient and pump were not altered between or during measurements (Fig 2). Inline pressure was measured for 20 s during relaxed breathing at each flow rate, and the maximal pressure was recorded. Clinical staff and participants were blind to the CRM results. These data were used to produce a pressure-flow scatter plot (Fig 3), and the gradient of this plot was estimated by a least squares regression line. This gradient was then used to represent the resistance of that lumen. Because the least squares regression line was assumed to represent laminar flow, results that were a poor fit to this line (*R* ^*2*^ <85%) were termed “non-laminar”, and resistance was not estimated.

Actual time-cost was estimated by measuring the time taken from initial access of the CVC until completion of CRM at or after the 5- and 10-week visits. This represents the time taken for CRM but does not include waiting or travel time.

### Assessment of acceptability

Acceptability was measured using a custom-designed acceptability questionnaire administered to the participant or the participant’s parent/caregiver if the participant was younger than 18 years, at or after the 4-, 5-, 11-, and 12-week visits (S1 and S2 Figs). The acceptability questionnaire was divided into 4 domains: participant-reported time-cost (3 items), physical adverse effects (3 items), psychological concerns (3 items), and overall acceptability (1 item). A total acceptability score was calculated by summing the scores of the first 3 domains and then adding triple the value of the overall acceptability score. The total acceptability score was expressed as a percentage of the maximum-possible score for all answered items. Acceptability data from the participants and caregivers were analyzed together.

### Statistical analysis

The gradient and coefficient of determination (*R* ^*2*^) of a pressure-flow scatter plot were calculated for each CRM visit. According to a perfect laminar flow model, the pressure-flow plot should produce a straight line that represents an estimate of the resistance of the entire system. The validity of this model depends on laminar flow; thus, CRM results that were a poor fit to a least squares regression line (*R* ^*2*^ <85%), were termed non-laminar, and resistance was not estimated for those visits. These results most likely represent turbulent flow. Non-laminar results were regarded as a potential predictor of occlusion and were therefore incorporated into the final predictive model.

**Fig 4:**
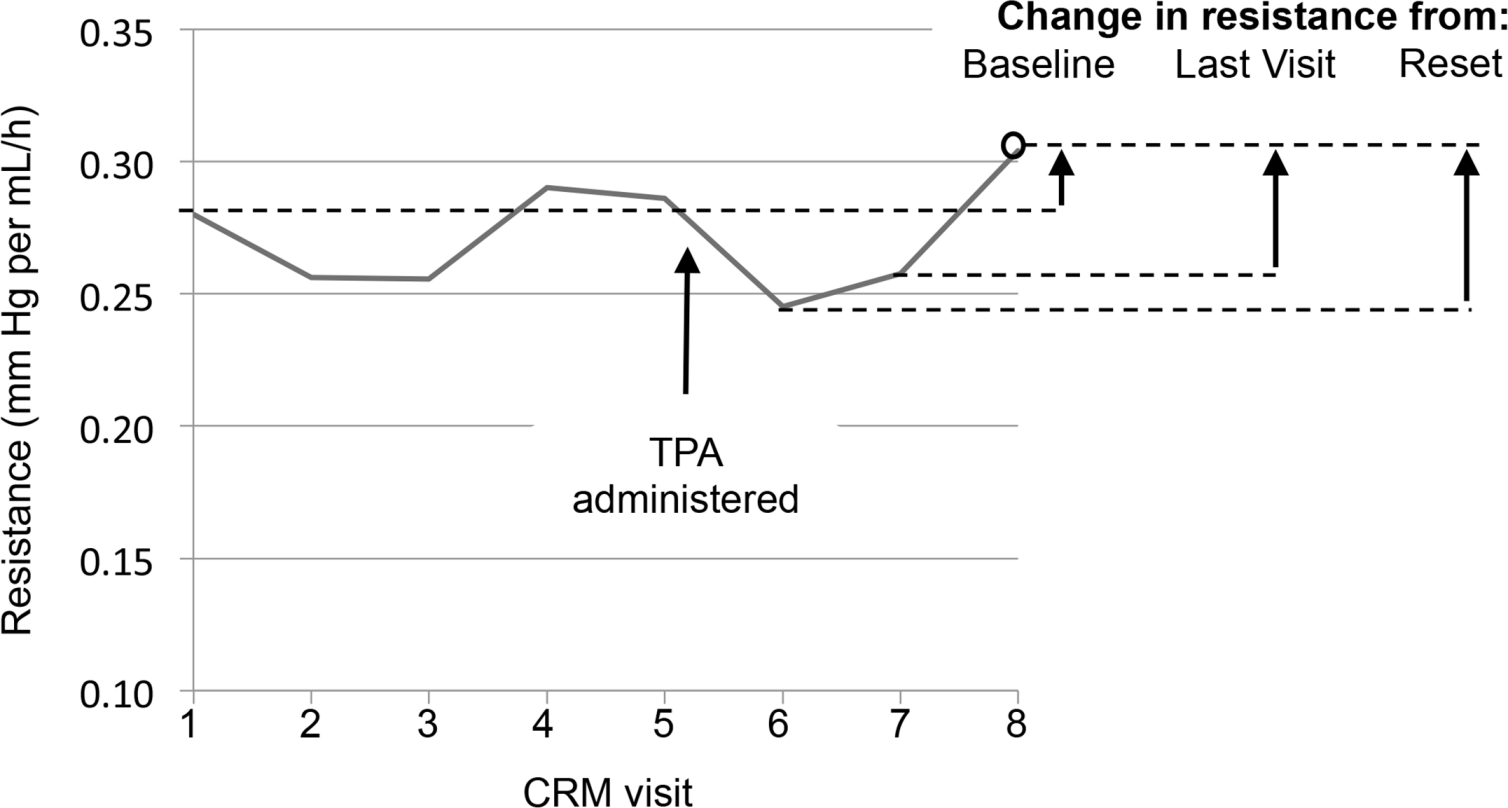
Metrics used to report change in resistance. Change in resistance at each visit was described as the proportional change in estimated resistance within each lumen compared to that at Baseline (i.e., enrollment or first CRM visit), Reset (i.e., first CRM visit after TPA administration), or Last Visit (i.e., immediately previous CRM visit). Figure shows data from a single study participant and catheter lumen.

A number of metrics were used to describe the change in resistance, including proportional changes in resistance within each lumen compared to that measured in the same lumen at the time of enrollment/first CRM (i.e., Baseline), at the first CRM after TPA administration (i.e., Reset), or at the previous CRM visit (i.e., Last Visit) (Fig 4).

A 2-sample Wilcoxon rank sum test was used to determine the significance of differences in changes in resistance between CRM visits that preceded occlusion events compared with those that did not, and Fisher’s exact test was used to assess all dichotomous outcomes. A receiver operating characteristic (ROC) curve was applied to describe the performance of CRM as a predictive test for CVC occlusion, and the Youden index was used to determine the cut-off that provided the most accurate results. Cronbach’s alpha was used to assess the reliability of the acceptability questionnaire, and a paired *t*-test was used to determine the significance of change in mean acceptability scores from earlier to later time points.

### Ethics Statement

This study was prospectively approved by the St. Jude Institutional Review Board as “Catheter resistance monitoring to predict adverse events in children and adolescents (CaRMA)” and registered with clinicaltrials.gov (NCT01737554), and a modification to allow early analysis due to the high occlusion rate seen was approved by the same committee. Potential participants were approached during hospitalization or clinic visits, and written informed consent was obtained from participants or their parents/caregivers, as appropriate. Gift cards valued at $5 were provided at each CRM visit to compensate for the participant’s time and effort. CRM was not performed if occlusion to flush was present at the time of the study visit.

## Results

Ten participants were enrolled and followed for a median of 15 weeks (range, 13–15 weeks). No eligible patients were approached but declined participation. The median number of CRM visits was 12 (range, 8–12). Two participants missed 1 or 2 visits, and 1 participant discontinued the study after 8 visits because displacement of the CVC necessitated its removal. Baseline demographic and CVC data (Table 1) and occurrence of clinically significant CVC-related complications (Table 2) are summarized below.

**Table 1:** Demographics and CVC specifications for 10 participants.

Characteristic	No. participants^a^
Mean age ± SD (years)	12.1 ± 4.1
Male sex	7
Primary diagnosis	
* Acute leukemia*	6
* Solid tumor*	2
* Nonmalignant hematologic disorder*	2
Bone marrow transplantation	4
Active malignancy	4
Median no. days since insertion (range)	23.5 (3–183)
No. lumens	
* Single*	1
* Double*	9
Subclavian location	10
Size	
* *7 Fr (0.8/1.0 mm ID)	4
* *9 Fr (0.7/1.3 mm ID)	5
* *9.6 Fr (1.6 mm ID)	1

^a^ Unless otherwise indicated, the data represent the number of participants.

**Abbreviations:** CVC, central venous catheter; Fr, French catheter gauge; ID, inner diameter of the lumen; No., number of; SD, standard deviation;

**Table 2:** CVC-related complications.

CVC complications	No. participants affected (n/1000 CVC days)
Occlusion events	33 (37.4)
* Total occlusion*	6 (6.8)
* Other*	27 (30.6)
Events requiring TPA	17 (19.3)
* Total occlusion*	5 (5.7)
* Other*	12 (13.6)
Venous thrombosis	0
CLABSI	1 (1.1)

**Abbreviations:** CLABSI, central line–associated bloodstream infection; CVC, central venous catheter; No., number of; TPA, tissue plasminogen activator

No adverse events associated with CRM were identified, and clinically apparent venous thrombosis (n = 0) and CLABSI (n = 1) were infrequent. However, occlusion was more common (n = 33), as were occlusion events requiring TPA therapy (n = 17). Correlation between CRM and occlusion events was, therefore, further analyzed.

**Fig 5:**
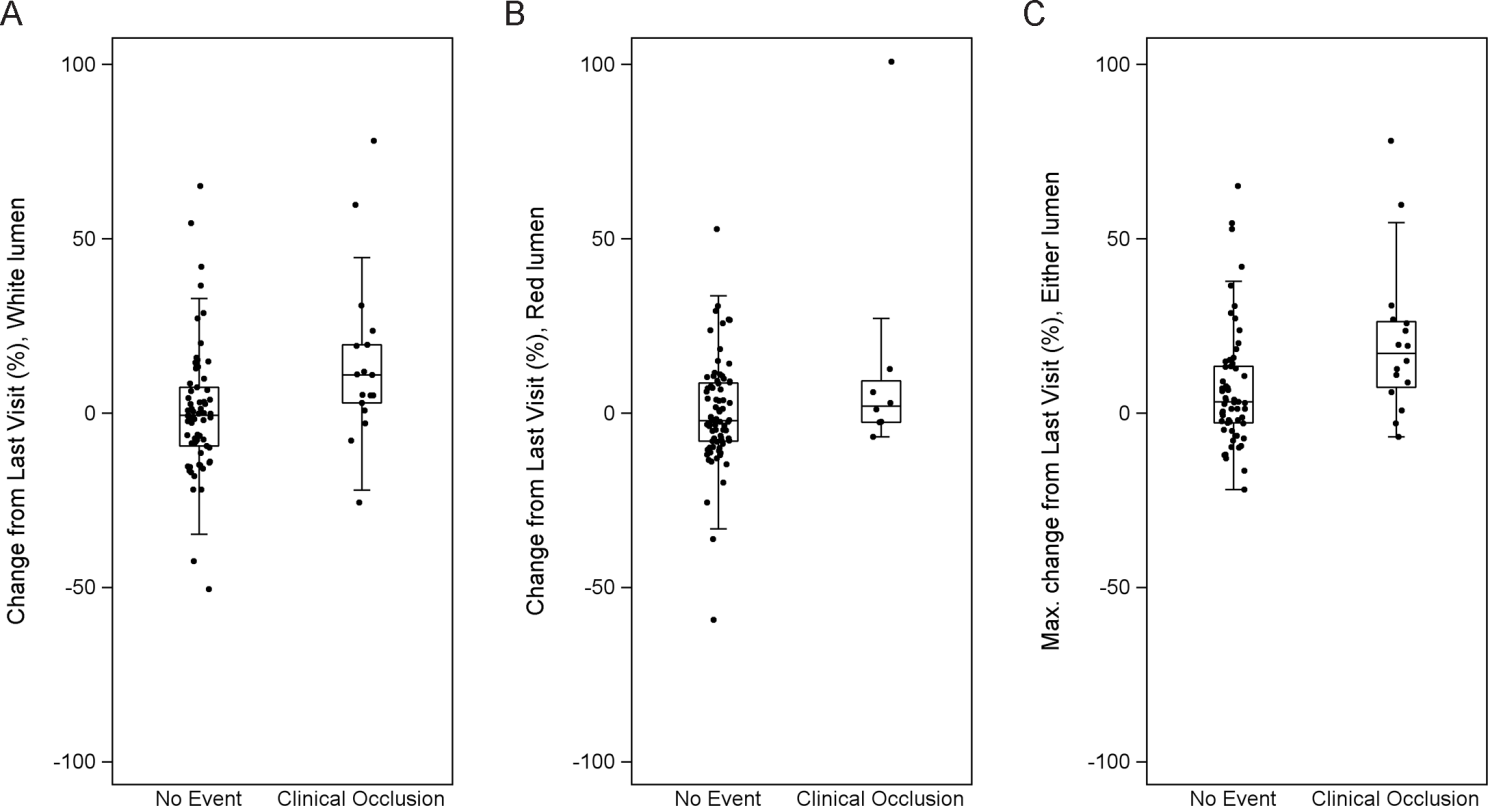
Scatter plot of catheter-resistance monitoring results. Results are stratified by whether a clinical occlusion occurred within 10 days. The changes in resistance from Last Visit are shown. (A) Change in resistance in the smaller white lumen only, (B) the red lumen only, or (C) maximal change in either lumen. Occlusion was frequently preceded by a detectable rise in resistance that was not clinically apparent.

A change in resistance of individual CVC lumens strongly predicted occlusion in the smaller white lumen (Table 3). For this lumen, median change in CVC resistance from Last Visit was significantly higher in participants who had a clinical occlusion event in the subsequent 10 days than it was in those who did not (10.9% vs. –0.7%; p = 0.01). The difference in change from Baseline was also significant (p = 0.02), but change from Reset was not (p = 0.2). In contrast, the larger red lumen showed a similar trend for change from Last Visit, but it was not significant (2.0% vs. –2.2%; p = 0.3) (Fig 5). A separate analysis of events that required thrombolytic therapy showed similar results (Table 3).

**Table 3:** Changes in CVC resistance preceding an occlusion within 10 days.

Lumen/Time interval	Any occlusion event			An occlusion event requiring TPA		
	Yes % change (range)	No % change (range)	P-value	Yes % change (range)	No % change (range)	P-value
White^a^						
* Last Visit*	10.9	–0.7	0.01	19.4	–0.2	0.01
	(–25.7, 78.1)	(–50.5, 65.2)		(–25.7, 78.1)	(–50.5, 65.2)	
* Reset*	1.0	–2.2	0.2	7.9	–2.4	0.1
	(–25.7, 178.8)	(–50.5, 64.0)		(–25.7, 178.8)	(–50.5, 64.0)	
* Baseline*	11.1	–2.0	0.02	7.9	–1.5	0.2
	(–57.2, 178.8)	(–55.0, 63.7)		(–57.2, 178.8)	(–55.0, 111.8)	
Red^a^						
* Last Visit*	2.0	–2.2	0.3	–0.7	–1.8	1.0
	(–6.7–100.9)	(–59.2, 52.8)		(–6.7, 6.0)	(–59.2, 100.9)	
* Reset*	1.8	2.0	1.0	0.2	2.0	0.5
	(–9.9, 193.1)	(–41.7, 159.6)		(–9.9, 7.1)	(–41.7, 193.1)	
* Baseline*	–2.8	2.3	0.4	–9.9	2.6	0.1
	(–20.5, 193.1)	(–41.7, 159.6)		(–20.5, 7.1)	(–41.7, 193.1)	
Either^b^						
* Last Visit*	17.1	3.3	0.01	19.6	3.6	0.02
	(–6.7, 78.1)	(–22.0, 65.2)		(–6.7, 78.1)	(–22.0, 65.2)	
* Reset*	7.1	7.4	0.2	22.0	6.8	0.03
	(–13.3, 178.8)	(–13.7, 64.0)		(1.7, 178.8)	(–13.7, 159.6)	
* Baseline*	7.1	7.4	0.2	14.6	7.1	0.1
	(–13.5, 178.8)	(–13.7, 63.7)		(–13.5, 178.8)	(–13.7, 159.6)	

^a^ Data represent the median proportional change in CVC pressure from the noted time point.

^b^ Data represent the median maximal proportional change in CVC pressure from the noted time point.

**Abbreviations:** CVC, central venous catheter; TPA, tissue plasminogen activator

Because events caused by external factors such as fibrin sheaths, malposition, or displacement might affect both lumens, we hypothesized that the maximal change in either lumen would be a good predictor of occlusion in either lumen. In fact, the maximal change in resistance in either lumen at each CRM visit did strongly predict subsequent occlusion in either lumen (Table 3). Median maximal change from Last Visit was significantly higher in participants who had a clinical occlusion within 10 days (17.1% vs. 3.3%; p = 0.01). Maximal changes from Baseline were not significantly different, and maximal change from Reset was significantly higher for visits preceding events that required treatment with TPA only.

**Fig 6:**
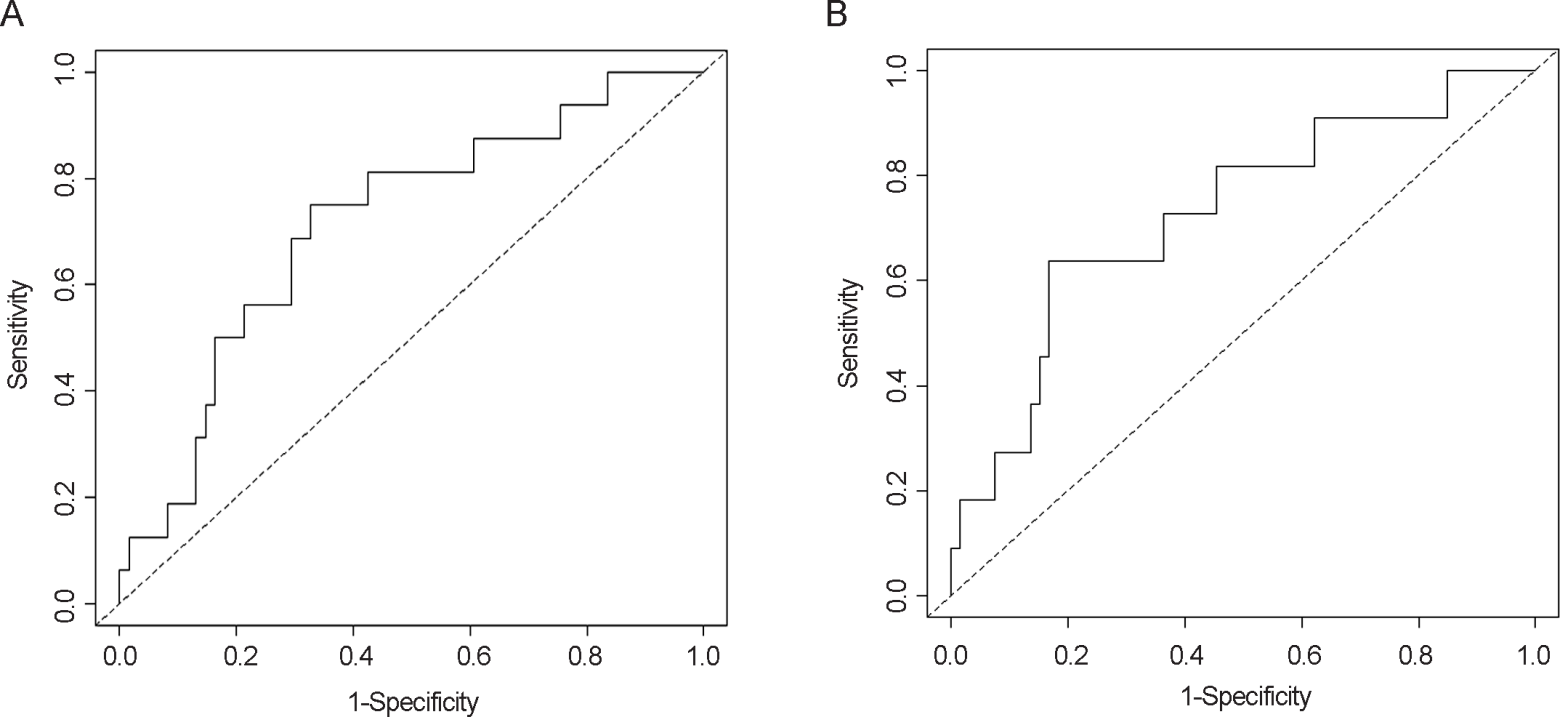
Receiver operating characteristic analysis for maximal change from Last Visit to predict catheter occlusion within 10 days. (A) Any occlusion event and (B) an occlusion event requiring TPA therapy.

ROC analysis showed that a maximal change of 8.8% from Last Visit best predicted occlusion within 10 days (p<0.01; sensitivity, 75%; specificity, 67%; AUC = 0.71) (Fig 6A). A higher cut-off of 19.2% best predicted occlusion events requiring treatment with TPA within 10 days (p = 0.01; sensitivity, 64%; specificity, 83%; AUC = 0.73) (Fig 6B). When the change of pressure from Last Visit was greater than 8.8%, the odds ratio of occlusion within 10 days was 6.2 (95% CI, 1.8–21.5).

Non-laminar results (*R* ^*2*^ <85%) were examined separately. Measuring resistance in each lumen independently, we categorized 9 measurements as non-laminar on 8 visits (1 participant had non-laminar results from both lumens during a single visit). The risk of an occlusion in the same lumen within 10 days was significantly greater after a non-laminar result (55.6% vs. 15.8%; p<0.01). Occlusion in either lumen within 10 days tended to be more frequent after a non-laminar result (50.0% vs. 24.8%; p = 0.2). On the basis of these findings, we created the following model: Visits that had a maximal change from Last Visit that was greater than 8.8% or a non-laminar result for at least 1 lumen were regarded as “positive tests.”

This model accounts for the predictive values of both the maximal change in resistance and non-laminar results. The risk of CVC occlusion within 10 days was significantly higher following a positive test (40.0% vs. 8.9%, p = 0.002). This model was the best predictor of CVC occlusion within 10 days (odds ratio = 6.8; 95% CI, 2.0–22.8; sensitivity, 80%; specificity, 63%). Data that were used in the model are shown in Fig 1.

The feasibility and acceptability of CRM were very high and did not change over the course of the study (Table 4). Time-cost was measured at 17 visits in 10 participants; mean time (± SD) for CRM was 26 ± 8.7 min/week. Thirty-six of 38 (95%) acceptability questionnaires were returned. Correlation between acceptability data collected on consecutive weeks showed that the questionnaire had very high test-retest stability (Cronbach alpha = 0.85). Participants rarely expressed concerns in any of the domains or overall, and the mean acceptability scores at the earlier and later time points did not differ (92.7% vs. 93.8%; p = 0.63).

**Table 4:** Feasibility and acceptability of catheter-resistance monitoring.

Feasibility and acceptability measures	Mean percentage ± SD
Feasibility measures	
* CRM visits attended* ^a^	96.4 ± 6.1
* CRM data obtained* ^b^	100.0 ± 0
Acceptability domains	
* Time-cost*	91.0 ± 10.2
* Psychological concerns*	93.1 ± 8.9
* Physical adverse events*	94.1 ± 8.7
* Overall acceptability*	92.7 ± 7.5

^a^ The data represent the proportion of planned CRM visits that the participants attended.

^b^ The data represent CRM visits during which catheter resistance measures were obtained for at least 3 flow rates in each CVC lumen.

**Abbreviations:** CRM, catheter-resistance monitoring; CVC, central venous catheter; SD, standard deviation

## Discussion

Central venous catheters often become occluded, and this event is linked to clinically significant adverse outcomes. As many as 20% of occlusion events require CVC removal, and attempts to clear occlusion can cause CVC rupture or fracture [1,6,9,16]. Even after successful treatment, the risk of CLABSI is significantly increased [10]. Occlusion events, especially recurrent occlusion, are also associated with subsequent venous thrombosis [1,8]. Furthermore, 2 studies in pediatric oncology patients found that individuals with CVC occlusion have higher all-cause mortality [7,8]. This work highlights the need to prevent occlusions by predicting their imminent occurrence and providing pre-emptive therapy.

Predicting occlusion has been investigated previously in 2 small studies. Stokes *et al*. prospectively examined catheter resistance in pediatric oncology patients. They found that resistance was often higher than predicted by in vitro experiments, even in the absence of clinical abnormalities (e.g., difficulty flushing or aspirating the CVC), and that treatment with thrombolytic agents (e.g., urokinase) decreased resistance [13]. The investigators concluded that subclinical CVC obstruction is common and might progress to a clinically significant event, but they did not routinely perform serial measurements or correlate their findings with clinical outcomes. In the second study, Arai *et al*. used inline pressure monitoring to detect occlusion of peripherally inserted central catheters (PICC) in neonates [17]. They found that the inline pressure in PICCs generally varies little over time, but occasional episodes of persistently increased pressure, attributed to partial occlusion, do occur. The investigators also reported a number of episodes of total occlusion due to thrombosis, kinking, or accidental compression by an incubator door. They did not note whether occlusion was preceded by measurable increases in pressure.

The current study demonstrates that serial monitoring of CVC resistance is feasible and acceptable in the pediatric hematology and oncology population. Satisfaction scores and adherence were high and did not deteriorate over the 12 weeks of study participation. CVC access was performed according to institutional standards and there were no adverse events detected. No participants experienced venous thrombosis and only one experienced CLABSI. Although the sample size is small, it is reassuring that the rate of CLABSI in this study (1 event in 942 patient days) is not greater than published data for this population [18,19]. Catheter occlusion occurred frequently enough to preliminarily assess the sensitivity and specificity of the new method. CRM performed well for predicting these events. Using ROC analysis, we selected a cut-off for test positivity to maximize sensitivity and specificity.

Despite its biological and clinical plausibility, CRM did not always successfully predict CVC occlusion. Some episodes of increased resistance did not lead to clinically apparent dysfunction; in those cases, the increased resistance appeared to be self-resolving. However, it is possible that events like these still increase the risk of subsequent venous thrombosis or CLABSI. Conversely, some occlusion events were not preceded by a significant increase in catheter resistance; those episodes might represent a different mechanism of occlusion, with a more rapid onset. They also suggest that more frequent monitoring may be required for maximal sensitivity. Importantly, there is evidence that bacterial biofilm can cause CVC occlusion with a very rapid onset due to biofilm streamers acting as a “sieve-like network” [5].

The current study does have some limitations. Firstly, to distinguish “non-laminar” CRM measures based on the laminar flow model, a cut-off of *R* ^*2*^>85% was applied. This cutpoint was chosen *a priori* as indicating an adequate fit to the proposed flow model. In a larger sample, other cut-off values could be explored using a sensitivity analysis to demonstrate how robust the results are to choice of *R* ^*2*^ cut-off. In contrast, rather than being predetermined, the cut-off for significance of change in resistance was selected by ROC analysis to be the best predictor of clinical events. This cut-off should, therefore, be prospectively evaluated in a confirmation cohort. Not all participants were able to attend every CRM visit or complete all questionnaires. Because venous imaging was performed only as clinically indicated, subclinical venous thrombosis events could have been missed, but none of the participants experienced a symptomatic venous thrombosis. The rate of occlusion was higher than expected; events included incomplete occlusion, simple dysfunction, and total occlusion, so variation in practice between treating clinicians may have affected this. Similarly, some TPA-treated events might have self-resolved if intervention had not occurred. It is important to note that treating clinicians were blind to the CRM results and study data did not influence the decision to treat.

The natural history of non-laminar CRM results is unknown. However, the increased rate of subsequent occlusion suggests that turbulent flow is a clinically significant phenomenon. This notion could be further investigated by immediate repetition of resistance measurements when non-laminar results are obtained in future studies. Whether acceptability and feasibility data obtained in this study are applicable to other pediatric populations or to adult patients is unknown. In this pilot study, we chose not to adjust for multiple testing which increased the risk of type I error; the findings reported in this paper should be confirmed with a larger study to better control for this.

Potential future research directions include streamlining the CRM process, assessing immediate reproducibility of non-laminar results and resistance measurements, determining the effect of patient position on measured resistance, assessing test-performance in larger diameter catheters, and investigating the clinical impact of pre-emptive thrombolytic therapy. Other risk factors for occlusion should be addressed in future prospective studies. The CRM approach is time consuming and technically challenging; developing and validating an automated system that modulates flow rate and measures pressure according to an algorithm similar to that reported here has the potential to allow convenient point-of-care testing to facilitate prompt intervention. An automated system would also provide an objective measure of catheter resistance without requiring specially trained staff. In the longer term, a larger prospective study of CRM-guided pre-emptive thrombolytic therapy is needed to identify clinically significant benefits beyond preventing occlusion.

## Conclusions

Catheter-resistance monitoring offers a feasible, noninvasive method for predicting CVC occlusion. The sensitivity and specificity of this approach are reasonable but imperfect. More work is needed to streamline the CRM process, determine the optimal frequency of testing, validate the intervention cut-offs, and evaluate the effect of pre-emptive therapy.

## Supporting Information

S1 Fig Acceptability questionnaire (Parent version). (PDF)

S2 Fig Acceptability questionnaire (Participant version). (PDF)

S3 Fig Resistance measurements for each participant visit, stratified by CVC lumen. Non-laminar results (*R* ^*2*^<85%) are excluded.
(TIF)

S1 File Initial IRB approval notice. Notice of protocol approval by the St. Jude Children’s Research Hospital Institutional Review Board.
(PDF)

S2 File Study Database. Database of results from each participant visit including: Study day at visit, measured pressure at each flow rate in each lumen, calculated resistance and coefficient of determination in each lumen, change in resistance form baseline and previous visit, most recent dose of thrombolytic agent, and days until each next line-related event in each lumen.
(XLSX)

S1 Protocol Study protocol as approved by the St. Jude Children’s Research Hospital Institutional Review Board.
(PDF)

S1 STARD Checklist Completed checklist of the Standards for Reporting of Diagnostic Accuracy Studies (STARD) initiative. (DOCX)
