# Supplementary material for: Monitoring Central Venous Catheter Resistance to Predict Imminent Occlusion: A Prospective Pilot Study
Source: PLoS One. 2015 Aug 31;10(8):e0135904. doi: 10.1371/journal.pone.0135904 (PMC4555832; doi:10.1371/journal.pone.0135904)
Supplement: S2 Fig — (PDF) [file pone.0135904.s002.pdf]

MR:
Date:
Study #:

Catheter Resistance Testing Questionnaire (Participant version)

Thank you for completing this short questionnaire about catheter resistance testing. There are 10 questions.

To answer each question, think about **the last two times** you had catheter resistance testing done.

You can leave comments if you want to.

Your answers will be placed in a sealed envelope and entered directly into a computer database by a staff member who is not directly involved in catheter resistance testing

1. Catheter resistance testing took up too much of my time

O	O	O	O	O	O
Strongly Disagree	Disagree	Somewhat Disagree	Somewhat Agree	Agree	Strongly Agree

Comments:

2. I was anxious or worried before catheter resistance testing

O	O	O	O	O	O
Strongly Disagree	Disagree	Somewhat Disagree	Somewhat Agree	Agree	Strongly Agree

Comments:

3. I had side-effects from catheter resistance testing

O	O	O	O	O	O
Strongly Disagree	Disagree	Somewhat Disagree	Somewhat Agree	Agree	Strongly Agree

Comments:

4. Catheter resistance testing interfered with other activities

O	O	O	O	O	O
Strongly Disagree	Disagree	Somewhat Disagree	Somewhat Agree	Agree	Strongly Agree

Comments:

MR:
Date:
Study #:

5. I felt calm during catheter resistance testing

○	○	○	○	○	○
Strongly Disagree	Disagree	Somewhat Disagree	Somewhat Agree	Agree	Strongly Agree

Comments:

6. Catheter resistance testing was uncomfortable or painful

○	○	○	○	○	○
Strongly Disagree	Disagree	Somewhat Disagree	Somewhat Agree	Agree	Strongly Agree

Comments:

7. Catheter resistance testing was easy to fit into my week

○	○	○	○	○	○
Strongly Disagree	Disagree	Somewhat Disagree	Somewhat Agree	Agree	Strongly Agree

Comments:

8. Catheter resistance testing was stressful

○	○	○	○	○	○
Strongly Disagree	Disagree	Somewhat Disagree	Somewhat Agree	Agree	Strongly Agree

Comments:

9. I didn't like the way catheter resistance testing felt

○	○	○	○	○	○
Strongly Disagree	Disagree	Somewhat Disagree	Somewhat Agree	Agree	Strongly Agree

Comments:

10. Overall, catheter resistance testing is acceptable to me

○	○	○	○	○	○
Strongly Disagree	Disagree	Somewhat Disagree	Somewhat Agree	Agree	Strongly Agree

Comments:
