# Supplementary figures and images for: Monitoring Central Venous Catheter Resistance to Predict Imminent Occlusion: A Prospective Pilot Study
Source: PLoS One. 2015 Aug 31;10(8):e0135904. doi: 10.1371/journal.pone.0135904 (PMC4555832; doi:10.1371/journal.pone.0135904)

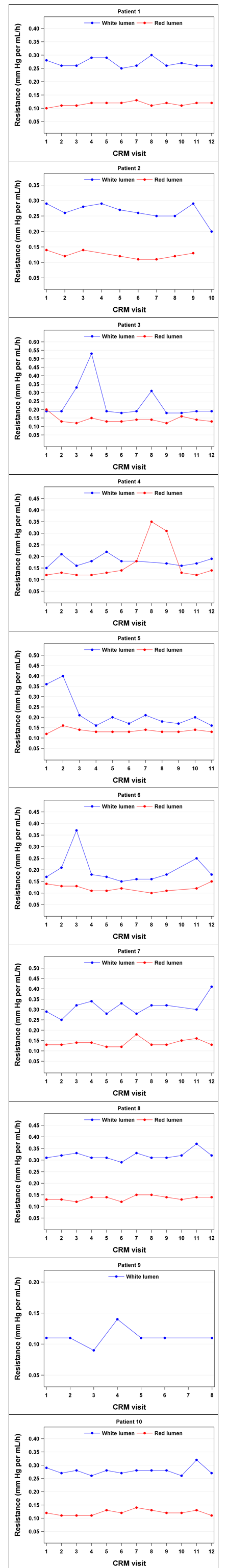

Supplement: S3 Fig — Non-laminar results (R 2<85%) are excluded. (TIF) [file pone.0135904.s003.tif]
