# Supplementary material for: Monitoring Central Venous Catheter Resistance to Predict Imminent Occlusion: A Prospective Pilot Study
Source: PLoS One. 2015 Aug 31;10(8):e0135904. doi: 10.1371/journal.pone.0135904 (PMC4555832; doi:10.1371/journal.pone.0135904)
Supplement: S1 Protocol — Jude Children’s Research Hospital Institutional Review Board. (PDF) [file pone.0135904.s006.pdf]

CATHETER RESISTANCE MONITORING TO PREDICT CATHETER-ASSOCIATED ADVERSE EVENTS IN CHILDREN AND ADOLESCENTS: A FEASIBILITY STUDY

Faculty Sponsor
Patricia M Flynn MD[1,2]

Principal Investigator
Joshua Wolf MBBS[1,2]

Co-Investigators
Jeffrey E Rubnitz[2,3]
David R Shook[4]
Karen D Wright[3]
Rachel C Brennan[3]
Dennis C Stokes[2,5]
Yimei Li[6]

1. Department of Infectious Diseases, St Jude Children's Research Hospital, Memphis, TN
2. Department of Pediatrics, University of Tennessee Health Science Center, Memphis, TN
3. Department of Oncology, St. Jude Children's Research Hospital, Memphis, TN
4. Department of Bone Marrow Transplantation & Cellular Therapy, St. Jude Children's Research Hospital, Memphis, TN
5. Division of Cardiopulmonary Diseases, St. Jude Children's Research Hospital, Memphis, TN
6. Department of Biostatistics, St Jude Children's Research Hospital, Memphis, TN

St. Jude Children's Research Hospital
262 Danny Thomas Place
Memphis, Tennessee 38105-3678
Telephone: (901) 595-3300

Contents of this document may not be extracted without permission from the Principal Investigator.

Protocol MNEMONIC and Title: CaRMA - Catheter Resistance Monitoring to predict catheter-associated Adverse events in children and adolescents: A feasibility study

Principal Investigator: Joshua Wolf MBBS

IND Holder: Not Applicable

Brief Overview:

Catheter occlusion and dysfunction are common complications of CVAD use in children with cancer and hematologic disorders. These events can lead to interruption of therapy and may require device removal and replacement. Attempts to clear occlusion can cause device fracture.

There is a clear link between catheter occlusion and other serious complications including bloodstream infection and intravascular thrombosis.

There is evidence that catheter occlusion or dysfunction may be preceded by subclinical catheter narrowing, which could be detected by accurate measurement of catheter resistance.

This study aims to describe the feasibility and results of catheter resistance monitoring (CRM) over time with the aim of prospectively identifying patients at high risk of catheter occlusion.

If CRM is feasible and proves to be sensitive and specific, it could provide an opportunity for pre-emptive therapy to prevent occlusion, which might also prevent bloodstream infection or thrombosis.

Intervention:

The intervention in this study is weekly measurement of catheter resistance. This will be determined using the Alaris® Syringe Module (Carefusion inc., San Diego, USA), a commercially available, FDA approved IV pump which is able to deliver accurate flow-rates and obtain accurate pressure measurements. The inline pressure will be measured at multiple flow-rates and resistance will be estimated from the gradient of the pressure-flow curve. Treating clinicians will be blind to the results, but no intervention or test is prohibited during the study.

Objectives:

Primary Objective:

To describe the feasibility of weekly CRM in children and adolescents treated at St. Jude

Secondary Objectives:

- To describe patient and caregiver adherence with weekly CRM in children and adolescents treated at St. Jude
- To explore the correlation between results of CRM and catheter occlusion or dysfunction

Exploratory Objectives:

- To assess acceptability of weekly CRM for participants and caregivers
- To measure the time-cost of CRM
- To explore the correlation between results of CRM and catheter-associated thrombosis
- To explore the correlation between results of CRM and central line associated bloodstream infection

Responsible Investigator for all objectives: Dr Joshua Wolf
Estimated date for completion of data collection: 11/01/2013

Hypotheses/Estimates:

Primary Hypothesis:

That CRM is feasible in children and adolescents treated at SJRCH

Secondary Hypotheses:
That adherence to weekly CRM will be high in children and adolescents treated at SJRCH

That clinically apparent catheter occlusion or dysfunction is preceded by a measurable rise in catheter resistance which is outside the range of baseline variability

Exploratory Hypotheses:

That weekly CRM will be highly acceptable to patients and caregivers

That the mean time required for CRM will be less than 15 minutes per lumen per visit

That clinically apparent catheter-associated thrombosis and CLABSI are preceded by a measurable rise in catheter resistance which is outside the range of baseline variability

Criteria for Evaluation:

The primary outcome measure will be the proportion of attended CRM visits for each participant for which usable catheter resistance measurements are obtained for each lumen of the CVAD.

Catheter resistance will be measured by determining the gradient of a pressure-flow curve over a range of clinically relevant flow-rates. These data will be collected weekly for each lumen of the CVAD by the PI or trained study staff.

Other outcome measures include: changes in resistance preceding clinical events, attendance at planned weekly CRM visits and patient/caregiver satisfaction scores.

Study Design: The design will be prospective single-group feasibility study

Study Population:

Inclusion Criteria:
1. Receiving treatment for any disease at SJRCH
2. Age ≥5 to <25 years
3. Single or double lumen tunneled CVAD (ports will not be eligible)
4. Participant anticipates being present weekly at SJRCH for at least 12 weeks

Exclusion Criteria:
1. Plan to remove CVAD within 12 weeks
2. Expected survival less than 12 weeks
3. Past enrollment in the CaRMA study or past catheter resistance monitoring

Sample Size: 35 evaluable participants

Data Analyses:

Planned Analyses for Primary Study Aim:

The proportion of attended weekly visits for each patient for which usable resistance data are obtained will be determined and reported as the primary outcome.

Planned Analyses for Secondary Aims:

Attendance at weekly visits over the study period for each participant will be expressed as a proportion of planned visits.

The change in catheter resistance from baseline over time will be described for each participant. Change from baseline for measurements preceding catheter occlusion or dysfunction events will be compared with other measurements.

Planned Analyses for Exploratory Aims:

Acceptability of the intervention will be determined by caregiver or participant

responses to a custom-built acceptability questionnaire administered at 4, 5, 11 and 12 weeks.

Time taken for up to two CRM visits per patient will be determined at, or after, the planned 5 and 10 week visits.

The change in catheter resistance from baseline over time will be described for each participant. Change from baseline for measurements preceding catheter-associated thrombosis and bloodstream infections will be compared with other measurements.

Primary Anticipated Completion Date: 10/11/13

Anticipated Study Completion Date: 11/01/13

Timeframe for Primary Outcome Measure: All outcomes will be reported for 15 weeks from entry to study or until 3 weeks after discontinuation of CRM, whichever is shorter.

Data Management: Data management will be provided locally by the Department of Infectious Diseases at St. Jude Children's Research Hospital.

Human Subjects: No significant risk to participants is expected. Increased catheter access could be associated with increased risk of infection, but the relative increase in risk is expected to be minimal as the catheters are routinely accessed and flushed at least once per lumen per day.

DEFINITIONS

Central venous access device (CVAD): An intravenous catheter that terminates at or close to the heart or in one of the great vessels which is used for infusion, withdrawal of blood, or hemodynamic monitoring

Catheter resistance monitoring: Serial measurement of resistance to flow in the lumen(s) of a CVAD with the aim of identifying an increase in resistance greater than normal variation.

Total CVAD occlusion: Inability to flush and aspirate any lumen of a CVAD which does not resolve with standard techniques. Standard techniques include: Checking that the CVAD is not clamped or kinked, flushing, changing position, raising arms or coughing.

CVAD occlusion to aspiration: Inability to aspirate but not flush any lumen of a CVAD which does not resolve with standard techniques as above.

CVAD occlusion to flush: Inability to flush but not aspirate any lumen of a CVAD which does not resolve with standard techniques as above.

CVAD dysfunction: Subjective increase in pressure required to flush or aspirate any lumen of a CVAD which does not resolve with standard techniques as above.

Intravascular thrombosis: Radiologically proven total or subtotal occlusion of a large vein in which a CVAD has been placed. Thrombosis will be categorized as symptomatic if any of the following are noted: extremity swelling, redness or pain, or if there is clinical evidence of pulmonary embolism.

Central line associated bloodstream infection: For the purposes of this study, CLABSI will be defined according to the National Healthcare Safety Network (NHSN) definition, modified to account for local practice. [Centers for Disease Control, 2010]

Laboratory confirmed BSI, not secondary to infection at another site, where a central line (intravascular catheter which terminates at or close to the heart, or in one of the great vessels) is present within 48 hours of infection onset

Laboratory-Confirmed BSI: A bloodstream infection which meets one of the following criteria:

Criterion 1: Patient has a recognized pathogen cultured from one or more blood cultures; And organism cultured from blood is not related to an infection at another site.

Criterion 2: Patient has at least one of the following signs or symptoms: fever (>38°C), chills, or hypotension; And signs and symptoms and positive laboratory results are not related to an infection at another site; And a common skin contaminant is cultured from two or more blood cultures drawn on separate occasions.

Criterion 3: Patient < 1 year of age has at least one of the following signs or symptoms: fever (>38°C core) hypothermia (<36°C core), apnea, or bradycardia; And signs and symptoms and positive laboratory results are not related to an infection at another site;

And a common skin contaminant is cultured from two or more blood cultures drawn on separate occasions.

Criterion 4*: Patient has at least one of the following signs or symptoms: fever (>38°C), chills, or hypotension; And signs and symptoms and positive laboratory results are not related to an infection at another site; And a common skin contaminant is cultured from a single blood culture; And the treating clinician elects to treat as CLABSI.

Criterion 5*: Patient < 1 year of age has at least one of the following signs or symptoms: fever (>38°C core) hypothermia (<36°C core), apnea, or bradycardia; And signs and symptoms and positive laboratory results are not related to an infection at another site; And a common skin contaminant is cultured from a single blood culture; And the treating clinician elects to treat as CLABSI.

*Criteria 4 and 5 are added to the original NHSN criteria to allow for local practice at St Jude in which a single set of blood cultures are routinely collected prior to initiation of systemic antibiotics.

Common skin contaminant: An organism which occurs naturally on the skin and is recognized as a common contaminant of blood cultures, but which can cause CLABSI (i.e., diphtheroids, *Bacillus spp*. [not *B. anthracis*], *Propionibacterium spp*., coagulase-negative staphylococci, viridans streptococci, *Aerococcus spp*., *Micrococcus spp*.)

Port: CVAD in which the entire device is implanted subcutaneously and accessed through the skin. Also called a subcutaneous port or totally implantable venous access device (e.g. Infusaport®, Port-A-Cath®)

Tunneled line: CVAD which has a subcutaneous tunnel and exits through the skin (e.g. Hickman®, Broviac® or Groshong® catheters)

Table of Contents

Study Summary

1.0 OBJECTIVES 1
1.1 Primary Objective..1
1.2 Secondary Objectives..1
1.3 Exploratory Objectives..1
2.0 BACKGROUND AND RATIONALE..1
2.1 Background 1
2.2 Rationale..7
3.0 RESEARCH PARTICIPANT ELIGIBILITY CRITERIA AND STUDY ENROLLMENT 8
3.1 Inclusion Criteria..9
3.2 Exclusion Criteria..9
3.3 Research Participant Recruitment and Screening 9
3.4 Enrollment on Study at St. Jude 9
4.0 DESIGN AND METHODS..10
4.1 Design and Study Overview..10
4.2 Baseline Data Collection..10
4.3 Measurement of Catheter Resistance 11
4.4 Results of Catheter Resistance Monitoring..11
4.5 Outcome Assessment 12
5.0 REQUIRED EVALUATIONS, TESTS, AND OBSERVATIONS 15
5.1 Pre-Study Evaluations 15
5.2 Evaluations during Study 15
6.0 OFF THERAPY AND OFF STUDY CRITERIA 15
6.1 Criteria for Discontinuation of Study Intervention 15
6.2 Off Study Criteria..16
7.0 SAFETY AND ADVERSE EVENT REPORTING REQUIREMENTS........16
8.0 DATA COLLECTION, STUDY MONITORING, AND CONFIDENTIALITY 17
8.1 Data Collection..17
8.2 Study Monitoring..17
8.3 Collection of Data from St. Jude Affiliates and Other Institutions 17
8.4 Confidentiality..17
9.0 STATISTICAL CONSIDERATIONS..18
9.1 Analysis of Primary Objective 18
9.2 Analysis of Secondary Objectives..18
9.3 Analysis of Exploratory Objectives..19
10.0 OBTAINING INFORMED CONSENT 19
10.1 Consent at Age of Majority..20
10.2 Consent When English is Not the Primary Language 20
11.0 REFERENCES 21

Appendices
Appendix I: Schedule of Events
Appendix II: Tests Performed for Good Clinical Care
Appendix III: Research Tests
Appendix IV: Acceptability questionnaire

1.0 OBJECTIVES

1.1 Primary Objective

To describe the feasibility of CRM in children and adolescents treated at St. Jude

1.2 Secondary Objectives

1.2.1 To describe patient and caregiver adherence with weekly CRM in children and adolescents treated at St. Jude

1.2.2 To explore the correlation between results of CRM and catheter occlusion or dysfunction

1.3 Exploratory Objectives

1.3.1 To assess acceptability of weekly CRM for participants and caregivers

1.3.2 To measure the time-cost of CRM

1.3.3 To explore the correlation between results of CRM and catheter-associated thrombosis

1.3.4 To explore the correlation between results of CRM and central line associated bloodstream infection

2.0 BACKGROUND AND RATIONALE

2.1 Background

Catheter occlusion is common

Catheter occlusion is a common and important adverse event. Inability to use the CVAD interrupts treatment and can require removal and replacement. Occlusion events requiring treatment occur at a rate of 2.0 - 2.2 per 1000 catheter days in pediatric oncology patients. [Journeycake, 2006; Dillon, 2004] A number of different mechanisms have been reported for CVAD occlusion. (Figure 1)

The rate of catheter occlusion events requiring thrombolytic intervention at St Jude appears similar to these published figures. In 2011 approximately 700 doses of recombinant tissue plasminogen activator (tPA) were distributed for treatment of CVAD occlusion. There are approximately 900 patients with catheters treated at St Jude at any time (~328,500 days per year). [Shenep, 2011] This gives an occlusion rate of

~2.2 per 1000 catheter days. However, this estimate does not take into account that some events may have required more than one dose of tPA (31.5% in one study). [Peng, 2011] Nor does it include occlusion events which were treated elsewhere or which led to catheter removal without intervention.

Figure 1. Mechanisms of catheter occlusion. [Stokes, 1989]

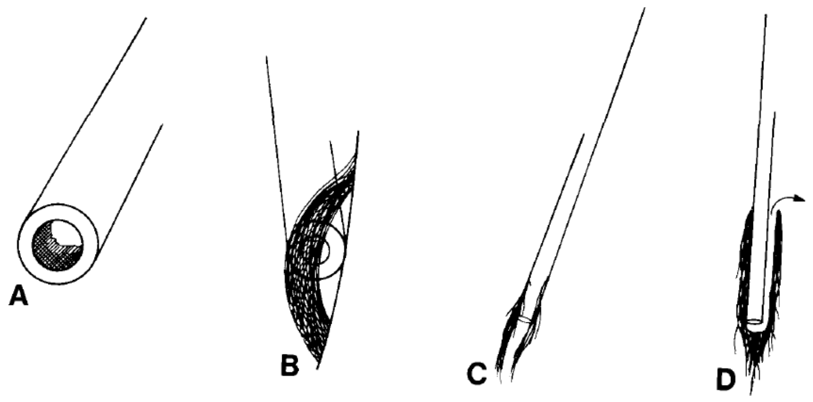

Treatment of catheter occlusion

Administration of a thrombolytic agent, such as tPA, commonly restores CVAD patency. However, at least 10% of episodes necessitate device removal [Journeycake, 2006], and catheter occlusion was responsible for 9.5% of unplanned catheter removals in a large pediatric oncology cohort. [Weiner, 1992]

One recent retrospective study of tPA use for treatment of catheter occlusion or dysfunction in a large children's hospital found that 21.3% of attempts were unsuccessful. [Peng, 2011] The study included patients with cancer (52.6%). There was a significant difference in failure rates between tunneled lines (19%) and ports (53.8%).

Catheter occlusion which is not amenable to thrombolytic treatment usually requires surgical management to replace the line.

Even for successful tPA use, the cost is significant. Each dose of tPA is currently $88, and St. Jude currently uses approximately 700 doses per year ($61,000).

Although tPA use is generally safe, and major bleeding occurs in less than 0.03% of treatment episodes, there are other potential drawbacks of attempted catheter salvage. [Baskin, 2009] In a study of children receiving antibody treatment for neuroblastoma, one of three patients who had complete catheter occlusion developed catheter fracture with

migration of the distal fragment into the right atrium. [Kayton, 2008] This is a rare but life-threatening complication of catheter occlusion.

Figure 2. Catheter fracture during attempted salvage of occluded catheter. [Kayton, 2008]

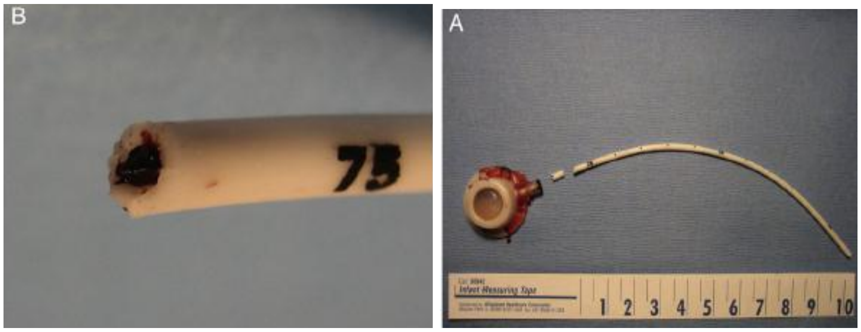

Catheter occlusion is related to infection and other complications

In addition to the morbidity directly associated with catheter occlusion, there is a complex relationship between catheter occlusion and the other two most frequent serious CVAD complications, infection and thrombosis. [Raad, 1994] Patients who have catheter occlusion are at higher risk of these events, and prevention of occlusion may prevent infection.

One prospective study of pediatric hematology and oncology patients with CLABSI found that 12.9% had an episode of catheter occlusion within 7 days prior to the event, compared with an expected rate of 1.4% based on published data. This finding is limited by the absence of a matched control group.

There is other good evidence that CLABSI is partly related to catheter occlusion. Three studies of techniques to prevent occlusion have found that the techniques had a significant impact on infection rates as well as the primary outcome. In a double-blind, randomized controlled trial of urokinase prophylaxis in children with cancer, urokinase reduced the risk of CLABSI by 27%. [Dillon, 2004] An observational study showed that systemic anticoagulation with low molecular weight heparin or vitamin K antagonists was associated with 54% fewer CLABSI episodes in children receiving long-term TPN. [Vegting, 2012] Monthly tPA prophylaxis was associated with a low rate of CLABSI in children with hemophilia who had implantable ports (0.04 episodes/1000 catheter days). [Jeng, 2009]

Catheter occlusion may be predictable by resistance measurement

Currently available data suggest that occlusion is rarely due to a sudden event, but more commonly follows slow accumulation of material which

eventually completely inhibits flow. [Stokes, 1989] A number of different mechanisms for catheter occlusion are shown in Figure 1 (above). [Stokes, 1989]

Stokes *et al* measured *in vitro* line resistance to infusion of normal saline in unused Hickman and Broviac catheters (expected resistance) and compared it to measured resistance in 18 patients with indwelling catheters. [Stokes, 1989] They found that patients with measured resistance significantly higher than expected resistance frequently had signs of early catheter occlusion by contrast imaging or response to urokinase instillation. They argued that measurement of line resistance could be used to guide intervention to prevent total occlusion. They did not routinely perform serial measurements in individual patients or seek to demonstrate the natural history of elevated line resistance. There were no adverse events associated with resistance measurements.

Arai *et al* used inline pressure monitoring to detect occlusion of peripherally inserted central catheters (PICC) in neonates. [Arai, 2002] They enrolled 50 neonates for 64 monitoring episodes (mean length of episode 11.7 days). They showed that there was minimal variation in pressure over time in PICCs which terminated in a central vein. There were some episodes of persistently increased pressure which were attributed to partial occlusion. They also reported a number of episodes of total occlusion due to thrombosis, kinking, or accidental compression by an incubator door. They did not report whether occlusion was preceded by measurable increases in line pressure.

Yang *et al* monitored the development of catheter occlusion in rats by serial measurement of resistance in CVADs. [Yang, 2005] They showed gradual increase in resistance over a number of weeks leading to almost inevitable occlusion or catheter dysfunction.

In a pig model of portal vein thrombosis, monitoring of mesenteric vein pressure has been trialed as a method of identifying partial occlusion with the aim of preventing total occlusion. [Yamataka, 1994]

Resistance measurement may be feasible

A number of studies have monitored resistance to flow in peripheral intravenous cannulae (PIVC) to detect infiltration or extravasation events. [Goodie, 1995; Scott, “Detection of intravenous fluid extravasation using resistance measurements”, 1996; Scott, “Resistance to fluid flow in veins”, 1996] These studies used measurement of flow at multiple different pressures to derive an estimate of resistance in the system. These studies demonstrated the practicality of measuring resistance using multiple flow-rates. The benefit of this process for predicting extravasation events was limited by large variation in pressure in subcutaneous tissues.

Resistance to flow can be estimated by measuring the relationship of pressure and flow-rate in the catheter lumen. Calculating catheter-resistance by a single measurement is inaccurate because it fails to account for central venous pressure. Instead, a number of pressure measurements are taken at different flow-rates and resistance is determined by the relationship between pressure and flow over the entire range. The resistance of the system is equal to the gradient of the pressure-flow curve. Figure 3 shows the relationship between pressure, flow and resistance.

Figure 3. Diagram of relationship between flow, pressure and resistance in CVAD

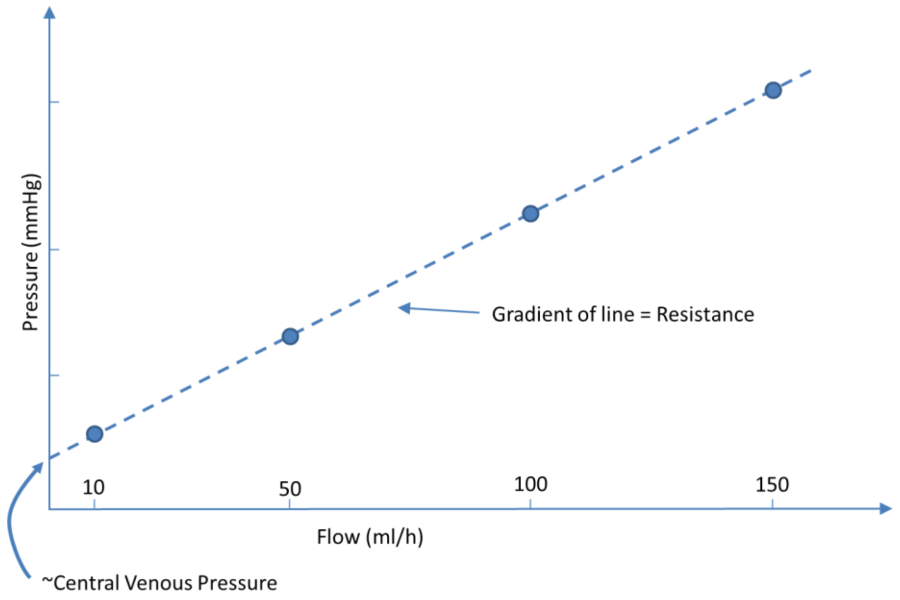

The Alaris® Syringe Module (Carefusion inc., San Diego, USA) is an FDA approved IV pump able to deliver very accurate fluid flow-rates (set rate +/- 2%). The device also has a sensitive and accurate inline pressure monitoring system using a 'pressure-sensing disc'. Because the device is able to accurately determine flow-rate and measure pressure, it provides reliable data about resistance to flow. Pressures generated by the device are equivalent to normal clinical conditions, so there is no increased risk of catheter damage.

We undertook preliminary *in vitro* experiments with this device to determine reproducibility of resistance measurements and the effect of back pressure (the equivalent of *in vivo* central venous pressure or bed-height) on measured resistance. In two separate experiments, resistance in a single catheter was measured at different flow-rates with the catheter tip at varying heights relative to the pump.

Figure 4 shows flow-pressure curves for each experiment.

Figure 4. Reproducibility of *in vitro* Catheter Resistance Monitoring

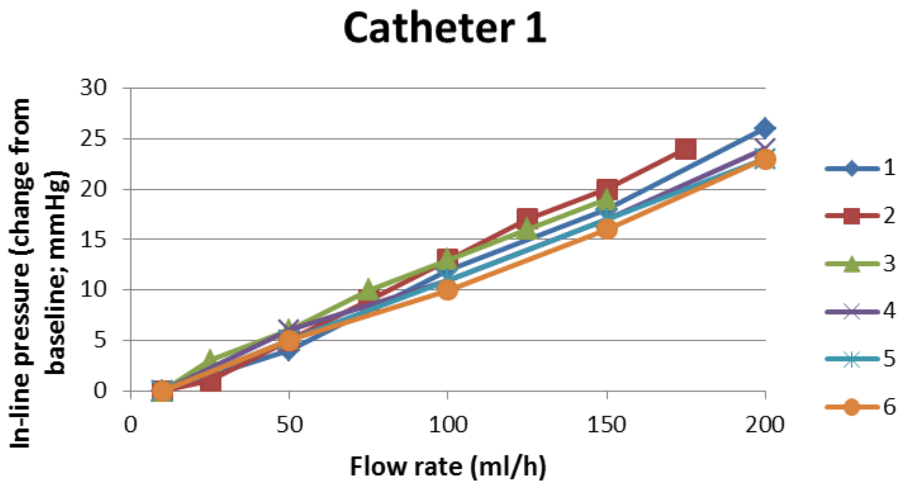

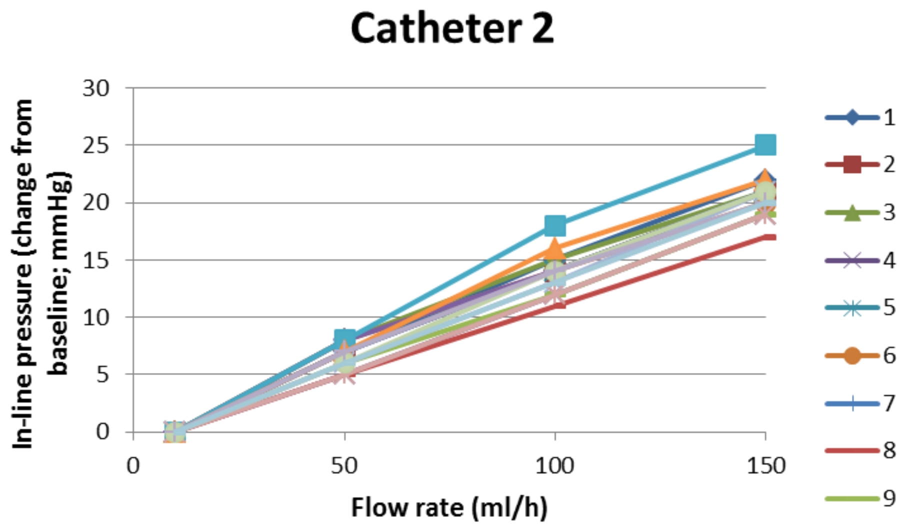

For Catheter 1, mean resistance was 0.13 mmHg/ml/hr (Range 0.12 – 0.15; standard deviation 0.012) over 6 trials. For Catheter 2, mean resistance was 0.15 (Range 0.12 – 0.18; standard deviation 0.012) over 9 trials. Variability was small (maximum change from mean 20%).

By measuring pressure at multiple flow-rates in an *in vitro* system with the catheter tip set at various heights relative to the pump we were able model the effect of repeated measurement and difference in CVP or bed height, and demonstrated that these had minimal effect on measured resistance. These data show that *in vitro*, resistance measurement is feasible and reproducible.

Serial monitoring of catheter-resistance in patients without clinical evidence of catheter occlusion could demonstrate that occlusion events or other CVAD-related complications are predictable, and suggest an ideal time-point or other marker to guide intervention.

2.2 Rationale

Catheter occlusion is an important complication of CVAD use in children. It has serious consequences, and is related to bloodstream infection and intravascular thrombosis which in turn can cause significant morbidity and mortality.

There is some evidence that measurement of line resistance in CVADs might predict the future development of catheter occlusion, that early intervention could prevent this, and that serial measurement of line resistance might guide the use of these interventions.

Catheter resistance monitoring will be performed using the Alaris® Syringe Module (Carefusion inc., San Diego, USA). The technique for resistance measurement involves use of the device at clinically relevant flow-rates and is expected to cause minimal inconvenience for participants and caregivers.

Occlusion occurs when line resistance rises beyond a threshold at which no flow can be produced at maximum safe pressure. The study aims to show that CRM can identify a rise in resistance before the threshold is crossed and before catheter dysfunction is clinically apparent. However, the technique remains exploratory as the baseline variability and rapidity of rise in resistance is unknown. A number of different patterns might emerge.

Figure 5 shows a diagram of possible resistance patterns leading to an inevitable occlusion event. Line A shows high baseline variability with an unpredictable course to occlusion. Line B shows a steady rise in resistance over time with minimal baseline variability and predictable eventual occlusion. Line C shows a very rapid rise in resistance immediately preceding an unpredictable occlusion event. Lastly, Line D shows a slower exponential rise in resistance with a potentially predictable occlusion event.

If Line A or C is eventually found to be descriptive of most *in vivo* events, CRM is unlikely to be of assistance due to high baseline variability or insufficient prior warning. Alternatively, Line B or D might make CRM a realistic option for assessing the need for pre-emptive intervention to prevent catheter occlusion.

Figure 5. Diagram of possible patterns of change in resistance over time preceding catheter occlusion.

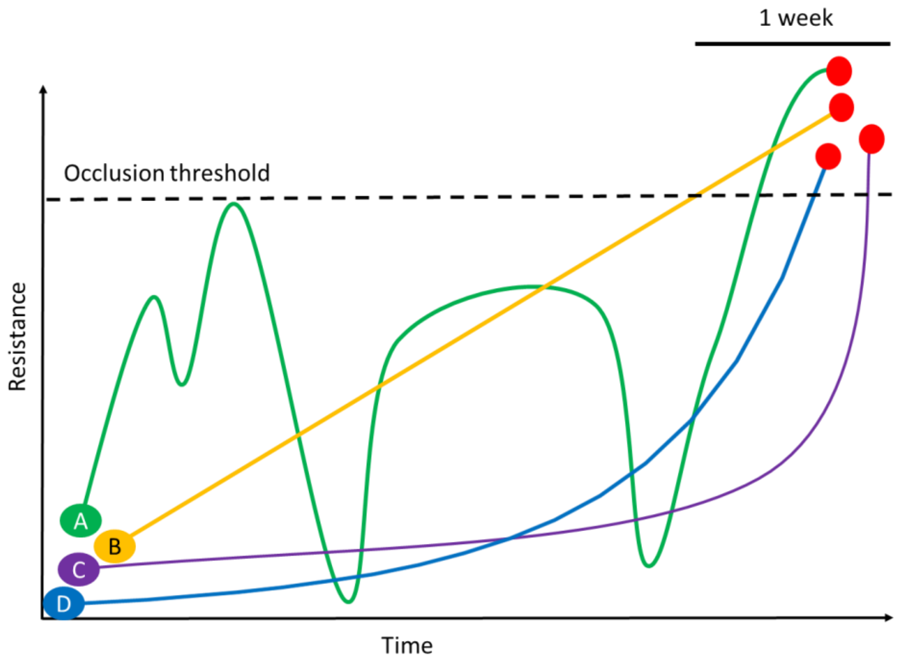

If the CRM technique is found to be feasible and predictive of future events, it will open the way for future research examining the use of pre-emptive intervention to prevent occlusion, dysfunction, thrombosis and infection. This could have a major impact on supportive care for children with cancer who require central venous access for survival. CRM is also a component of ETHEL, another St. Jude initiated study which aims to determine the impact of ethanol lock therapy on the outcome of CLABSI.

For this study, only children and adolescents expected to be present at St Jude for assessment at least weekly will be eligible. However, if the technique is shown to be successful in the St Jude population, other populations may also benefit. Children with intestinal insufficiency receiving long-term TPN are at extremely high risk of catheter associated complications. Catheter removal and replacement is extremely serious for these children as exhaustion of potential CVAD sites can lead to death or intestinal transplantation. [Kaufman, 2001]

3.0 RESEARCH PARTICIPANT ELIGIBILITY CRITERIA AND STUDY ENROLLMENT

According to institutional and NIH policy, the study will accession research participants regardless of gender and ethnic background. Institutional experience confirms broad representation in this regard.

3.1 Inclusion Criteria

3.1.1 Receiving treatment for any disease at SJRCH

3.1.2 Age ≥5 years to <25 years

3.1.3 Single or double lumen tunneled CVAD (ports will not be eligible)

3.1.4 Participant anticipates being present weekly at SJRCH for at least 12 weeks

3.2 Exclusion Criteria

3.2.1 Plan to remove CVAD within 12 weeks

3.2.1 Expected survival less than 12 weeks

3.2.3 Past enrollment in the CaRMA study or past catheter resistance monitoring

Females who are capable of becoming pregnant, or are breastfeeding, and males who plan to father children will all be eligible for enrollment. No pregnancy test will be required.

3.3 Research Participant Recruitment and Screening

Research participants will be recruited from outpatient clinics and inpatient wards at SJRCH only.

Each recruitment day, a study staff member will approach treating clinicians to ask which patients might be appropriate for enrollment, based on the described inclusion and exclusion criteria. After obtaining permission from clinicians, potential participants will be approached by study staff and invited to enroll in the study.

3.4 Enrollment on Study at St. Jude

A member of the study team will confirm potential participant eligibility as defined in Section 3.1-3.2, and complete and sign the ‘Participant Eligibility Checklist’. The study team will enter the eligibility checklist information into the Patient Protocol Manager (PPM) system. Eligibility will be reviewed, and a research participant-specific consent form and assent document (where applicable) will be generated. The complete signed consent/assent form(s) must be faxed to the CPDMO to complete the enrollment process.

A log of eligible patients who declined enrollment and reasons for non-enrollment will be maintained. Catheter resistance monitoring is a component of ETHEL, another St. Jude initiated study examining the impact of ethanol lock therapy on outcomes of CLABSI however, none of the first five study participants eligible to participate in the CRM component chose to do so. With respect to the current study, if none of the first six, or less than two of the first 12 potential participants approached agree to participate, the study will be paused to review and, if possible, address the reasons for non-enrollment.

The CPDMO is staffed 7:30 am-5:00 pm CST, Monday through Friday. A staff member is available by pager Saturday, Sunday, and holidays from 8:00 am to 5:00 pm.

4.0 DESIGN AND METHODS

4.1 Design and Study Overview

The trial will be a single-group prospective study of weekly catheter resistance monitoring for 12 weeks.

Participants will be enrolled at any point during treatment.

The study aims to recruit 35 evaluable participants. Based on estimates by representatives of each hospital team, approximately 80 patients per year would be eligible for enrollment (Bone marrow transplant, 40; Leukemia, 10; Neurooncology, 15; Solid tumor, 15).

During the study period, participants will be monitored for CVAD related complications, adherence to CRM visits and participant/caregiver satisfaction.

Participants who receive thrombolytic therapy to restore CVAD patency, or whose CVAD is removed and replaced with another eligible tunneled CVAD will remain on study. The next CRM reading will act as a new baseline for each lumen.

4.2 Baseline data collection

Age, gender, primary disease type, remission status, treatment protocol and treatment week will be recorded at enrollment.

Catheter type, anatomic location, insertion date and current function will be recorded for the current catheter. Number of previous catheters within 12 months prior to enrollment and reasons for their removal will be recorded. Episodes of CLABSI, line occlusion or intravascular

thrombosis, and use of thrombolytic agents within 6 months prior to enrollment will be recorded.

4.3 Measurement of Catheter-Resistance

Catheter-resistance in each lumen of the CVAD will be measured weekly by study staff with competencies in line care. This will occur in the medicine room or inpatient floor at St. Jude.

1. The CVAD will be accessed according to usual institutional practice. This will occur immediately after collection of laboratory tests where possible to standardize the procedure. No clave/needleless connector will be used.

2. Subjective catheter function will assessed by flushing and aspirating the CVAD and recorded on a case report form.

3. Normal saline will be administered at a predetermined constant flow-rate by the Alaris® Syringe Module (Carefusion inc., San Diego, USA). The pressure sensor will be placed at the estimated height of the right atrium (5cm below the sternal angle).

4. Pressure will be measured using the Alaris® Syringe Module (Carefusion inc., San Diego, USA) and will be recorded on a case report form.

5. The process will be repeated twice at 4 different flow-rates (usually 10ml/h, 50ml/h, 100ml/h, 150ml/h) for each lumen. Only flow-rates within the normal range for the population will be used. Figure 6 shows expected results for this procedure.

6. The line will be flushed and locked according to usual institutional practice.

If total occlusion of one or both lumens is identified at step 2, CRM will not be performed on involved lumens until patency is achieved.

It is anticipated that the CRM procedure will take 10-20 minutes per lumen to complete.

At each weekly visit, participants will be offered a $5 gift card or other item of similar value.

4.4 Results of Catheter Resistance Monitoring

CRM results will be considered usable if pressure readings are recorded for at least 3 different flow-rates for each lumen of the CVAD.

Results of catheter resistance measurement will not be communicated to the participant or treating team because interpretation is currently unclear and this is not a routine part of standard care.

The serial measurements of catheter resistance will be used to describe baseline variability for all participants. CRM results preceding catheter-related complications will be compared with all other results.

Figure 6. Chart of expected results of measurement of line pressure at different flow-rates to determine resistance

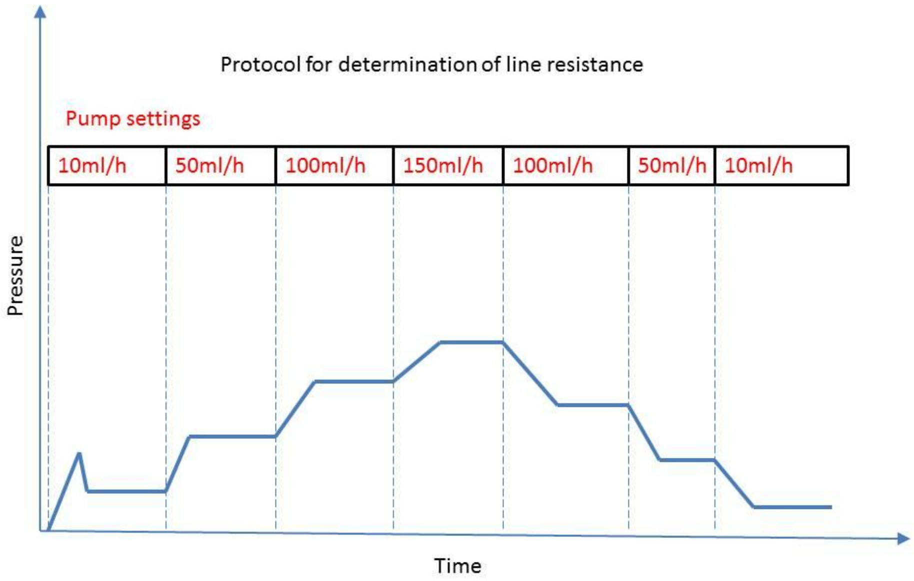

4.5 Outcome Assessment

Primary Objective

Feasibility of CRM will be determined by describing the proportion of attended CRM visits for each patient which produce usable resistance data (i.e. any pressure reading for at least 3 predetermined flow rates for each lumen of the CVAD). Feasibility will only be measured at attended visits to allow discrimination between feasibility and adherence which is assessed separately.

Secondary Objectives

Adherence

Attendance at each planned CRM visit will be recorded. An opportunity will be offered to make-up missed visits within one week of the due date – attendance at a make-up visit would count towards adherence for that week. The reasons provided for missed visits will be recorded. Adherence will be reported as proportion of planned weekly attended for each patient.

The total number of planned weekly visits will be 12 for all participants except those who discontinue the study intervention early due to death, transfer of care or permanent catheter removal. The number of planned visits will be capped at discontinuation date for these participants. For the purpose of the adherence analysis, participants who discontinue the study intervention early due to parent or clinician request will be assumed to have 12 planned visits.

Correlation between CRM results and catheter occlusion or dysfunction

At each weekly visit, information will be collected about worst line function in the past week. Data will be collected throughout the study for use of thrombolytic agents. Events will be confirmed where possible by examination of the medical record or direct contact with treating clinicians.

The relationship between CRM measurements and subsequent occlusion or dysfunction will be explored graphically.

All occlusion or dysfunction events which are managed with thrombolytic agents, or lead to long-term modification of CVAD use or CVAD removal, will be counted towards catheter occlusion or dysfunction. The type of event (total, aspiration or flush occlusion or dysfunction) will be determined by study staff after consultation with the treating clinical team.

Exploratory Objectives

Acceptability

Measurement of acceptability for medical interventions other than medication is not well standardized. [Atkinson, 2005]

Some investigators have used study retention and adherence as a surrogate of acceptability, but this has limitations. Although acceptability would be expected to partially predict attrition or non-adherence, perfect adherence does not necessarily represent perfect acceptability, especially in a highly motivated population with institutional loyalty such as leukemia patients at St Jude. In this population, families might even comply with an intervention which they find objectionable. Similarly, adherence with an acceptable intervention can be poor because of many factors unrelated to

acceptability. Other investigators have used questionnaires to assess acceptability, but there is currently no well-validated instrument.

A custom-built 10 item acceptability questionnaire focused on relevant domains of ‘acceptability’ will be used as the primary measure of this construct. A 6 point forced-choice Likert-type scale will be used for each item. (Appendix II)

The acceptability questionnaire will be administered at 4, 5, 11 and 12 weeks. The questionnaire will be completed at each time point by either the caregiver, or the participant if over 18 years of age. The completed questionnaires will be placed in a sealed opaque envelope and scored by a person other than the PI to encourage frank responses. Participants who discontinue the study intervention early will complete an acceptability questionnaire at time of exit from the study if possible, but will not complete further questionnaires after that time.

The proposed domains (subscales) of acceptability are; time-cost (Questions 1, 4 and 7), worry or anxiety (Questions 2, 5 and 8) and adverse effects (Questions 3, 6 and 9). There will also be a single item assessing ‘overall acceptability’ (Question 10).

Although the perception of necessity and efficacy are likely to be important correlates of acceptability of an intervention, it is incongruous to test these in the context of a study which provides no potential benefit to participants.

Scoring of the questionnaire will be performed as follows: Subscale scores will be determined by summing the scores for each item (1 - 6 points per item). Unanswered items will be excluded. The total acceptability score will then be determined by summing the score for each of the acceptability subscales plus three times the overall acceptability score and expressing this as a percentage of the maximum possible score for answered items.

Validity of the questionnaire will be assessed during the study by measuring reproducibility, convergent validity and predictive validity using simple regression analysis.

Predictive validity of the questionnaire will be determined by correlation between acceptability scores and adherence to subsequent study visits. Although not all non-adherence is related to non-acceptability, it would be expected that a correlation would exist if the questionnaire truly measures this construct.

Data will be collected throughout the study for diagnosis of bloodstream infection, intravascular thrombosis or pulmonary embolism. Events will be

confirmed where possible by examination of the medical record or direct contact with treating clinicians.

Radiology, microbiology and pharmacy records will be searched for confirmatory information about thrombosis, positive blood cultures and thrombolytic agent use. Information will be sought from affiliate hospitals and other sites for events which are reported to have occurred elsewhere.

5.0 REQUIRED EVALUATIONS, TESTS, AND OBSERVATIONS

The Schedule of Evaluations is shown in Appendix I.

For the duration of study participation, participants will be actively followed by review of the medical record, regular contact with the participant, and contact with pharmacy and the clinical microbiology laboratory for thrombolytic administration, new CLABSI and other catheter-related complications.

No laboratory tests will be performed for the study.

5.1 Pre-Study Evaluations

At study entry, baseline data will be collected as described in Section 4.2.

5.2 Evaluations During Study

Catheter resistance will be measured weekly during the study period as described in Section 4.3.

Data will be collected for catheter-related complications throughout the study period, until 21 days after the last CRM visit, as described in Section 4.5.

At weeks 4, 5, 11, and 12 (+/- 1 week) caregivers (or participants over the age of 18) will complete a questionnaire regarding acceptability of the CRM procedure. [Appendix II]

6.0 CRITERIA FOR REMOVAL FROM PROTOCOL AND OFF-STUDY CRITERIA

6.1 Criteria for Discontinuation of Study Intervention

6.1.1 Formal request of the Patient/Guardian to discontinue intervention
6.1.2 Formal request of the primary clinician to discontinue intervention
6.1.3 Permanent CVAD removal
6.1.4 Participant no longer attends St Jude for treatment or follow-up
6.1.5 Completion of 12 week intervention period

6.2 Off Study Criteria

6.2.1 Death
6.2.2 Permanent loss to follow-up
6.2.3 Formal request of the Patient/Guardian to discontinue intervention and data collection
6.2.4 Formal request of the primary clinician to discontinue intervention and data collection
6.2.5 Completion of all study requirements

7.0 SAFETY AND ADVERSE EVENT REPORTING REQUIREMENTS

Principal investigators are responsible for promptly reporting to the IRB any adverse events attributed to the intervention that are unanticipated, serious, and that may represent potential harm or increased risk to research participants. If an unanticipated study-intervention-related death occurs, the PI should report it to the Director of the Office of Human Subjects' Protection immediately upon becoming aware of the event by phone: (901) 595-4359, Cell: (901) 336-2894, fax: (901) 595-4361, or e-mail: hsp-1@stjude.org).

A reportable event entry into TRACKS will follow within 48 hours. Serious, unanticipated, study-intervention-related events must be reported within 10 working days.

In this study, adverse events related to cancer, cancer therapy and presence of a CVAD are expected. These include CLABSI, catheter occlusion and thrombosis. Adverse events directly attributable to the study intervention will be recorded according to CTCAE v4.0 and reported by the investigators.

The following definitions apply:

A **serious event** refers to any event in which the outcome is fatal or life-threatening, results in permanent disability, causes inpatient hospitalization or prolongs existing inpatient hospitalization, or is a congenital anomaly, cancer, or overdose.

An **unanticipated adverse event** refers to those not identified in their nature, severity, or frequency in the current risk documents (e.g. investigator's brochure), or consistent with the investigational plan.

The following are considered reportable: Any injuries, serious event or other unanticipated adverse events involving risk to participants or others which occur at a frequency above that considered acceptable by the investigators and the IRB. The OHSP Director or designee performs the initial review of unanticipated problems or serious adverse event reports. Internal reports of events that are unanticipated, serious, and related or possibly related to study interventions or procedures are then forwarded to the IRB Chair or designee and if necessary,

referred to the full IRB. Based on the frequency and seriousness of adverse events, the IRB Chair or Committee may deem it necessary to suspend or terminate a research study or studies.

8.0 DATA COLLECTION, STUDY MONITORING, AND CONFIDENTIALITY

8.1 Data Collection

The St. Jude data manager will review CRFs for accuracy and completeness. Data will then be entered into a secure database.

8.2 Data Collection Instructions for Affiliate Sites and Other Institutions

Study staff will collect information from St. Jude affiliates and other healthcare institutions as necessary.

8.3 Study Monitoring

Monitoring of this protocol is considered to be in the low risk category.

The Principal Investigator and study team are responsible for ensuring protocol compliance. The study team will hold quarterly team meetings and review case histories or quality summaries on participants.

Source document verification of eligibility and the informed consent process for 100% of St. Jude participants will be performed by The Eligibility Coordinators.

The Clinical Research Monitors will review up to 10% of the study participants annually for appropriateness of the informed consent process, eligibility, SAE reporting (TRACKS), and patient protocol status. Additional information may be monitored at the request of the Internal Monitoring Committee (IMC), the IRB, or other institutional administration. The Monitor will generate a formal report which is shared with the Principal Investigator (PI), study team and the IMC.

Continuing reviews by the IRB and CT-SRC will occur at least annually. In addition, SAE reports in TRACKS (Total Research and Knowledge System) will be reviewed in a timely manner by the IRB/OHSP.

8.4 Confidentiality

Study numbers and medical record numbers will be used in place of participant names in study documentation. No research participant names will be recorded on the stored data collection forms. The list containing

the study number and the medical record number will be maintained in a locked file and will be destroyed after all data have been analyzed.

The medical records of study participants may be reviewed by the St. Jude IRB, FDA, clinical research monitors, etc.

9.0 STATISTICAL CONSIDERATIONS

9.1 Analysis of Primary Objective

To describe the feasibility of CRM in children and adolescents treated at St. Jude

The proportion of attended CRM visits which produce usable resistance data for all lumens of the CVAD will be recorded for each participant, and summary statistics, including median, range and standard deviation will be reported for the study population.

Estimated completion date: 11/01/2013

9.2 Analysis of Secondary Objectives

9.2.1 To describe patient and caregiver adherence with weekly CRM in children and adolescents treated at St. Jude

Attendance at each planned CRM visit will be recorded. Summary statistics for proportion of planned visits attended for each participant, including median, range and standard deviation will be reported for the study population.

The total number of planned weekly visits will be 12 for all participants except those who discontinue the study intervention early due to death, transfer of care or catheter removal. The number of planned visits will be capped at date of death, transfer and catheter removal respectively for these participants. For the purpose of the adherence analysis, participants who discontinue the study intervention early due to parent or clinician request will be assumed to have 12 planned visits.

9.2.2 To explore the correlation between the results of CRM and catheter occlusion or dysfunction in children and adolescents

The relationship between CRM results and occlusion or dysfunction will be explored graphically as in Figure 5. Logistic regression models may be used to quantitate the correlation.

Occlusion events requiring treatment have been shown to occur at a rate of 2.0 - 2.2 per 1000 catheter days in pediatric oncology patients in previous

studies. [Journeycake, 2006; Dillon, 2004] Therefore, approximately 8 episodes of occlusion or dysfunction are expected during the study period.

9.3 Analysis for Exploratory Objectives

9.3.1 To assess acceptability of weekly CRM for participants and caregivers

To assess acceptability over the entire study period, mean acceptability scores for each participant from all timepoints will be calculated and described. To assess change in acceptability over the study period, mean acceptability scores for all patients at each timepoint will also be calculated and described.

The test-retest reliability of the acceptability questionnaire will be assessed using the intra-class correlation for each subscale and the overall acceptability score for questionnaires completed one week apart.

Predictive validity of the acceptability questionnaire will be explored using a logistic regression model to assess whether higher acceptability scores are associated with adherence at subsequent planned visits.

9.3.2 To measure the time-cost of weekly CRM

Time taken for up to two CRM visits for each patient will be recorded and reported. Visits on week 5 and 10 for each participant will be timed. These visits have been selected as a convenience sample as they will be near the middle and end of the study. If participants do not attend these visits, the next attended visit will be timed. Time will be measured from when the line is accessed until it is flushed or de-accessed. Summary statistics such as mean, range and standard deviation of time taken will be reported.

9.3.3 To explore the correlation between results of CRM and catheter-associated thrombosis or CLABSI in children and adolescents

The relationship between CRM results and thrombosis or CLABSI will be explored graphically as in Figure 5 and in 9.2.2. Logistic regression models may be used to quantitate the correlation.

10.0 OBTAINING INFORMED CONSENT

Eligible patients will first be approached by the patient’s primary physician or a member of the study team regarding the study purpose, methods and design details.

Both verbal and written assent and consent procedures will be completed in a private room and following St. Jude Children's Research Hospital institutional

guidelines. Verbal assent will be obtained from participants 7 to less than 14 years old and written assent from participants 14 to less than 18 years old. The consent/assent process will be documented in the medical record per institutional guidelines.

Research participants and parents may decline participation without any negative repercussions. Declinations will be documented in the research records and examined for any possible patterns.

If the informed consent process occurs on a different day to study entry, a discussion to assure continued interest in participation will occur at the time of screening and will be documented in the study database.

All patients who meet eligibility criteria regardless of gender or minority status are fully eligible to participate in this study. All data will be kept confidential and stored in locked offices.

10.1 Consent at Age of Majority

The age of majority in the state of Tennessee is 18 years old. Research participants will be consented at the next clinic visit after their 18th birthday.

10.2 Consent When English is Not the Primary Language

When English is not the patient, parent, or legally authorized representative's primary language, the Social Work department will determine the need for an interpreter. This information will be documented in the study database. Either a certified interpreter or the telephone interpreter's service will be used to translate the consent information. The process for obtaining an interpreter and for the appropriate use of an interpreter is outlined on the Interpreter Services, OHSP, and CPDMO websites.

11.0 REFERENCES

Arai J, Mouri Y, Miyamoto Y. Detection of peripherally inserted central catheter occlusion by in-line pressure monitoring. Paediatr Anaesth 2002;12:621-4.

Atkinson MJ, Kumar R, Cappelleri JC, Hass SL. Hierarchical construct validity of the treatment satisfaction questionnaire for medication (TSQM version II) among outpatient pharmacy consumers. Value in health : the journal of the International Society for Pharmacoeconomics and Outcomes Research 2005;8 Suppl 1:S9-S24.

Baskin JL, Pui CH, Reiss U, et al. Management of occlusion and thrombosis associated with long-term indwelling central venous catheters. Lancet 2009;374:159-69.

Centers for Disease Control and Prevention. National Healthcare Safety Network Patient Safety Component. 2010. (Accessed August 11, 2011, at http://www.cdc.gov/nhsn/psc.html.)

Dillon PW, Jones GR, Bagnall-Reeb HA, Buckley JD, Wiener ES, Haase GM. Prophylactic urokinase in the management of long-term venous access devices in children: a Children's Oncology Group study. J Clin Oncol 2004;22:2718-23.

Goodie DB, Philip JH. Is the i.v. obstructed or infiltrated? A simple clinical test. J Clin Monit 1995;11:47-50.

Jeng MR, O'Brien M, Wong W, et al. Monthly recombinant tissue plasminogen activator administration to implantable central venous access devices decreases infections in children with haemophilia. Haemophilia 2009;15:1272-80.

Journeycake JM, Buchanan GR. Catheter-related deep venous thrombosis and other catheter complications in children with cancer. J Clin Oncol 2006;24:4575-80.

Kayton ML, Garmey EG, Ishill NM, et al. Preliminary results of a phase I trial of prophylactic ethanol-lock administration to prevent mediport catheter-related bloodstream infections. J Pediatr Surg 2010;45:1961-6.

Peng C, Monagle P, Newall F. Clinical outcomes of management of CVAD occlusions. Archives of disease in childhood 2011;96:885-7.

Raad I, Luna M, Khalil SA, Costerton JW, Lam C, Bodey GP. The relationship between the thrombotic and infectious complications of central venous catheters. JAMA 1994;271:1014-6.

Scott DA, Fox JA, Cnaan A, et al. Resistance to fluid flow in veins. J Clin Monit 1996;12:331-7.

Scott DA, Fox JA, Philip BK, et al. Detection of intravenous fluid extravasation using resistance measurements. J Clin Monit 1996;12:325-30.

Stokes DC, Rao BN, Mirro J, Jr., et al. Early detection and simplified management of obstructed Hickman and Broviac catheters. J Pediatr Surg 1989;24:257-62.

Vegting IL, Tabbers MM, Benninga MA, et al. Prophylactic Anticoagulation Decreases Catheter-Related Thrombosis and Occlusion in Children With Home Parenteral Nutrition. JPEN J Parenter Enteral Nutr 2012.

Wiener ES, McGuire P, Stolar CJ, et al. The CCSG prospective study of venous access devices: an analysis of insertions and causes for removal. J Pediatr Surg 1992;27:155-63; discussion 63-4.

Yamataka A, Kawamoto S, Ishikawa M, et al. Indwelling mesenteric venous catheterization for early detection of portal vein thrombosis: possible application to pediatric liver transplantation. J Pediatr Surg 1994;29:58-60.

Yang J, Maarek JM, Holschneider DP. In vivo quantitative assessment of catheter patency in rats. Lab Anim 2005;39:259-68.

APPENDIX I

SCHEDULE OF EVALUATIONS:

		Catheter Resistance Monitoring Phase			Early study discontinuation
	Screen/ Entry	Week 1 to 12	Week 4, 5, 11, and 12 (±1 week)	Week 13-15	
Informed Consent	X				
History, height and weight[1]	X				
Interval History		X		X	X[a]
Satisfaction questionnaire		X	X		X
Resistance measurement		X			

1. Height and weight will be abstracted from the medical record where possible.
a. Interval history will be collected until 3 weeks after discontinuation of CRM if this occurs before 12 weeks.

APPENDIX II

TESTS PERFORMED FOR GOOD CLINICAL CARE

No tests or evaluations for this study are considered standard of care.

APPENDIX III

RESEARCH TESTS

The catheter resistance measurement and the satisfaction questionnaire are considered research.

APPENDIX IV

IVa: Acceptability questionnaire – parent version
IVb: Acceptability questionnaire – participant version

Catheter Resistance Testing Questionnaire (Parent version)

Thank you for completing this short questionnaire about catheter resistance testing. There are 10 questions.

To answer each question, think about **the last two times** you had catheter resistance testing done.

You can leave comments if you want to.

Your answers will be placed in a sealed envelope and entered directly into a computer database by a staff member who is not directly involved in catheter resistance testing

1. Catheter resistance testing took up too much of my time

O	O	O	O	O	O
Strongly Disagree	Disagree	Somewhat Disagree	Somewhat Agree	Agree	Strongly Agree

Comments:

2. My child appeared nervous or worried (e.g. fidgety, sweaty palms) before catheter resistance testing

O	O	O	O	O	O
Strongly Disagree	Disagree	Somewhat Disagree	Somewhat Agree	Agree	Strongly Agree

Comments:

3. My child had side-effects from catheter resistance testing

O	O	O	O	O	O
Strongly Disagree	Disagree	Somewhat Disagree	Somewhat Agree	Agree	Strongly Agree

Comments:

4. Catheter resistance testing interfered with other activities

O	O	O	O	O	O
Strongly Disagree	Disagree	Somewhat Disagree	Somewhat Agree	Agree	Strongly Agree

Comments:

5. My child was calm during catheter resistance testing

O	O	O	O	O	O
Strongly Disagree	Disagree	Somewhat Disagree	Somewhat Agree	Agree	Strongly Agree

Comments:

6. Catheter resistance testing was uncomfortable or painful

O	O	O	O	O	O
Strongly Disagree	Disagree	Somewhat Disagree	Somewhat Agree	Agree	Strongly Agree

Comments:

7. Catheter resistance testing was easy to fit into my week

O	O	O	O	O	O
Strongly Disagree	Disagree	Somewhat Disagree	Somewhat Agree	Agree	Strongly Agree

Comments:

8. Catheter resistance testing seemed stressful for my child

O	O	O	O	O	O
Strongly Disagree	Disagree	Somewhat Disagree	Somewhat Agree	Agree	Strongly Agree

Comments:

9. My child didn’t like the way catheter resistance testing felt

O	O	O	O	O	O
Strongly Disagree	Disagree	Somewhat Disagree	Somewhat Agree	Agree	Strongly Agree

Comments:

10. Overall, catheter resistance testing is acceptable to me

O	O	O	O	O	O
Strongly Disagree	Disagree	Somewhat Disagree	Somewhat Agree	Agree	Strongly Agree

Comments:

Catheter Resistance Testing Questionnaire (Participant version)

Thank you for completing this short questionnaire about catheter resistance testing. There are 10 questions.

To answer each question, think about **the last two times** you had catheter resistance testing done.

You can leave comments if you want to.

Your answers will be placed in a sealed envelope and entered directly into a computer database by a staff member who is not directly involved in catheter resistance testing

1. Catheter resistance testing took up too much of my time

O	O	O	O	O	O
Strongly Disagree	Disagree	Somewhat Disagree	Somewhat Agree	Agree	Strongly Agree

Comments:

2. I felt nervous or worried before catheter resistance testing

O	O	O	O	O	O
Strongly Disagree	Disagree	Somewhat Disagree	Somewhat Agree	Agree	Strongly Agree

Comments:

3. I had side-effects from catheter resistance testing

O	O	O	O	O	O
Strongly Disagree	Disagree	Somewhat Disagree	Somewhat Agree	Agree	Strongly Agree

Comments:

4. Catheter resistance testing interfered with other activities

O	O	O	O	O	O
Strongly Disagree	Disagree	Somewhat Disagree	Somewhat Agree	Agree	Strongly Agree

Comments:

5. I felt calm during catheter resistance testing

Strongly Disagree	Disagree	Somewhat Disagree	Somewhat Agree	Agree	Strongly Agree

Comments:

6. Catheter resistance testing was uncomfortable or painful

Strongly Disagree	Disagree	Somewhat Disagree	Somewhat Agree	Agree	Strongly Agree

Comments:

7. Catheter resistance testing was easy to fit into my week

Strongly Disagree	Disagree	Somewhat Disagree	Somewhat Agree	Agree	Strongly Agree

Comments:

8. Catheter resistance testing was stressful for me

Strongly Disagree	Disagree	Somewhat Disagree	Somewhat Agree	Agree	Strongly Agree

Comments:

9. I didn’t like the way catheter resistance testing felt

Strongly Disagree	Disagree	Somewhat Disagree	Somewhat Agree	Agree	Strongly Agree

Comments:

10. Overall, catheter resistance testing is acceptable to me

Strongly Disagree	Disagree	Somewhat Disagree	Somewhat Agree	Agree	Strongly Agree

Comments:
