# Supplementary material for: Monitoring Central Venous Catheter Resistance to Predict Imminent Occlusion: A Prospective Pilot Study
Source: PLoS One. 2015 Aug 31;10(8):e0135904. doi: 10.1371/journal.pone.0135904 (PMC4555832; doi:10.1371/journal.pone.0135904)
Supplement: S1 File — Notice of protocol approval by the St. Jude Children’s Research Hospital Institutional Review Board. (PDF) [file pone.0135904.s004.pdf]

Institutional Review Board #29
FWA00004775

11/8/2012

Joshua Wolf, MD
Clinical Education & Training

RE: CARMA - Catheter Resistance Monitoring to Predict Catheter-Associated Adverse Events in Children and Adolescents: A Feasibility Study

Dear Dr. Wolf,

This is to certify that, on 11/7/2012, the
New prospective study including protocol dated 9/24/2012 (document date 10/31/2012) and consent form dated 9/24/2012
submitted to the Institutional Review Board for consideration was reviewed by an IRB member using expedited procedures with respect to the adequacy of protecting the rights and welfare of participants, the use of appropriate methods of securing informed consent, the measures to be taken to minimize risk and the degree of risk relative to the potential benefits of the proposed research.

IRB Review Status:
This study is approved for the period of one year under 45CFR46.110(b)(1), Research Categories #s 4 and 7, and under children's category 45CFR46.404.

The study will be open to accrual upon activation by CPDMO.

IRB Approval Date: 11/7/2012
IRB Expiration Date: 11/7/2013

For further assistance, please contact the Office of Human Subjects' Protection at 901-595-4357 or email hsp-1@stjude.org.

(Submission Link: Pro00003392)

Reminder of Principal Investigator's Responsibilities:

As previously signed and certified, approval of this research involving human subjects is contingent upon your agreement:

1. To report to the Institutional Review Board for Human Research (IRB) any adverse effect or research related injuries which might occur in relation to the human experimentation. To read and comply with IRB reporting guidelines.
2. To submit in writing for prior IRB approval any alterations to the plan of human research.
3. To submit timely continuing review reports of this research as requested by the IRB.
4. To maintain copies of all pertinent information related to the research activities in this project, including copies of informed consent agreements obtained from all participants.
5. To notify the IRB immediately upon the termination of this project, and/or the departure of the principal investigator from this institution and the project.

Warning: This is a private message for Click Commerce clients & prospects only. If the reader of this message is not the intended recipient you are hereby notified that any dissemination, distribution or copying of this information is STRICTLY PROHIBITED.

St Jude Children's Research Hospital
Memphis, Tenn.
